# MACE and Hyperthyroidism Treated With Medication, Radioactive Iodine, or Thyroidectomy

**DOI:** 10.1001/jamanetworkopen.2024.0904

**Published:** 2024-03-04

**Authors:** Carol Chiung-Hui Peng, Yu-Jie Lin, Sun Y. Lee, Shu-Man Lin, Cheng Han, Ching-Hui Loh, Huei-Kai Huang, Elizabeth N. Pearce

**Affiliations:** 1 Section of Endocrinology, Diabetes, Nutrition and Weight Management, Boston University Chobanian and Avedisian School of Medicine, Boston, Massachusetts; 2 Diabetes Technology Center, Hualien Tzu Chi Hospital, Buddhist Tzu Chi Medical Foundation, Hualien, Taiwan; 3 School of Medicine, Tzu Chi University, Hualien, Taiwan; 4 Health Information Center, Tzu Chi University, Hualien, Taiwan; 5 Department of Physical Medicine and Rehabilitation, Hualien Tzu Chi Hospital, Buddhist Tzu Chi Medical Foundation, Hualien, Taiwan; 6 Department of Clinical Nutrition and Metabolism, Affiliated Zhongshan Hospital of Dalian University, Dalian, Liaoning, China; 7 Center for Healthy Longevity, Hualien Tzu Chi Hospital, Buddhist Tzu Chi Medical Foundation, Hualien, Taiwan; 8 Department of Family Medicine, Hualien Tzu Chi Hospital, Buddhist Tzu Chi Medical Foundation, Hualien, Taiwan; 9 Department of Medical Research, Hualien Tzu Chi Hospital, Buddhist Tzu Chi Medical Foundation, Hualien, Taiwan

## Abstract

**Importance:**

Excessive thyroid hormones from hyperthyroidism increase cardiovascular risks. Among 3 available treatments for hyperthyroidism, comparisons of long-term outcomes associated with antithyroid drugs (ATDs), radioactive iodine (RAI), and surgery to treat newly diagnosed hyperthyroidism are lacking.

**Objective:**

To compare risks of major adverse cardiovascular events (MACE) and all-cause mortality among patients with hyperthyroidism treated with ATDs, RAI, or surgery.

**Design, Setting, and Participants:**

This nationwide cohort study used the Taiwan National Health Insurance Research Database. Patients aged 20 years or older with newly diagnosed hyperthyroidism between 2011 and 2020 were enrolled. Treatment groups were determined within 18 months from diagnosis, with follow-up until the development of MACE, death, or the end date of the database, whichever came first. Data were analyzed from October 2022 through December 2023.

**Exposures:**

The ATD group received ATDs only. RAI and surgery groups could receive ATDs before treatment. Anyone who underwent thyroid surgery without RAI was classified into the surgery group and vice versa.

**Main Outcomes and Measures:**

The primary outcomes included MACE (a composite outcome of acute myocardial infarction, stroke, heart failure, and cardiovascular mortality) and all-cause mortality.

**Results:**

Among 114 062 patients with newly diagnosed hyperthyroidism (mean [SD] age, 44.1 [13.6] years; 83 505 female [73.2%]), 107 052 patients (93.9%) received ATDs alone, 1238 patients (1.1%) received RAI, and 5772 patients (5.1%) underwent surgery during a mean (SD) follow-up of 4.4 (2.5) years. Patients undergoing surgery had a significantly lower risk of MACE (hazard ratio [HR] = 0.76; 95% CI, 0.59-0.98; *P* = .04), all-cause mortality (HR = 0.53; 95% CI, 0.41-0.68; *P* < .001), heart failure (HR = 0.33; 95% CI, 0.18-0.59; *P* < .001), and cardiovascular mortality (HR = 0.45; 95% CI, 0.26-0.79; *P* = .005) compared with patients receiving ATDs. Compared with ATDs, RAI was associated with lower MACE risk (HR = 0.45; 95% CI, 0.22-0.93; *P* = .03). Risks for acute myocardial infarction and stroke did not significantly differ between treatment groups.

**Conclusions and Relevance:**

In this study, surgery was associated with lower long-term risks of MACE and all-cause mortality, while RAI was associated with a lower MACE risk compared with ATDs.

## Key Points

**Question:**

What are the long-term major adverse cardiovascular events (MACE) and all-cause mortality outcomes comparing the use of antithyroid drugs (ATDs), radioactive iodine (RAI), and surgery to treat newly diagnosed hyperthyroidism?

**Findings:**

In this cohort study of 114 062 patients with newly diagnosed hyperthyroidism, surgery was associated with a 24% lower risk of MACE and 47% lower risk of all-cause mortality, while RAI was associated with a 55% lower risk of MACE compared with ATDs.

**Meaning:**

These findings suggest that surgery or RAI may be better treatment options than long-term ATD use in patients with hyperthyroidism who are at risk of MACE.

## Teaser

This cohort study investigates the association of antithyroid drug, radioactive iodine, and surgery treatments with major adverse cardiovascular events in patients with newly diagnosed hypothyroidism.

## Introduction

Hyperthyroidism is characterized by excess thyroid hormone synthesis. Excessive circulating thyroid hormone enhances β-adrenergic activity and directly stimulates thyroid hormone receptors on myocardial and vascular endothelial tissues.^1,2^ Increasing evidence has demonstrated associations between hyperthyroidism and adverse cardiovascular outcomes, including atrial fibrillation, acute myocardial infarction (AMI), heart failure, stroke, and mortality.^1,2,3,4,5,6,7,8^

Hyperthyroidism can be treated with antithyroid drugs (ATDs), radioactive iodine (RAI) ablation, or surgery, which each have unique indications and safety profiles.^9,10^ Hyperthyroidism treatment approaches, particularly for Graves disease, have varied globally.^11^ ATDs have been the first-line therapy in Europe, Asia, and the Middle East.^12,13,14,15^ In the US, RAI was historically preferred,^12^ but ATD use has increased and is now the most commonly used primary treatment.^16^

The lack of population-level evidence comparing long-term benefits and risks of the 3 treatment modalities presents a challenge for informed decision-making and likely contributes to variations in treatment. In a 2021 cohort study,^17^ patients who received surgery within 12 months of diagnosis had dramatically lower risks of all-cause mortality and other cardiovascular diseases than patients treated with ATDs or RAI within 12 months of diagnosis. Atrial fibrillation risk was highest in patients treated with ATDs, followed by RAI and surgery in a Korean, population-based cohort.^18^ Other large cohort studies assessing cardiovascular morbidity and mortality have compared only 2 of the 3 available treatments.^19,20,21,22^

Based on current evidence, whether surgery or RAI should be preferred over ATDs for lowering major adverse cardiovascular events (MACE) and all-cause mortality remains uncertain. We aimed to assess long-term MACE and all-cause mortality among patients with newly diagnosed hyperthyroidism treated with ATDs, RAI, or surgery in a large Taiwanese database.

## Methods

### Data Sources

This nationwide cohort study used the Taiwan National Health Insurance Research Database (NHIRD), covering inpatient and outpatient records between 2011 and 2020. The NHIRD encompasses Taiwan’s entire population of approximately 23.6 million individuals, and details were previously described (eAppendix 1 in Supplement 1).^23,24^ We used the National Register of Deaths to confirm participant deaths. The Hualien Tzu Chi Hospital Institutional Review Board, which approved this study, also deemed that informed consent was not required because data were deidentified. We followed the Strengthening the Reporting of Observational Studies in Epidemiology (STROBE) reporting guideline for observational studies.

### Study Population, Exposure, and Follow-Up Time

Patients aged 20 years or older with newly diagnosed hyperthyroidism were identified using validated *International Classification of Diseases, Ninth Revision *(*ICD-9*) or *International Statistical Classification of Diseases and Related Health Problems, Tenth Revision *(*ICD-10*) codes for hyperthyroidism.^8,16,17,20,25^ The same diagnosis code had to occur twice in the inpatient or outpatient setting between 2011 and 2020 to confirm each patient’s diagnosis. Eligible codes for Graves disease, toxic nodular disease, and unspecified hyperthyroidism are listed in eTable 1 in Supplement 1. Patients were divided into ATD, RAI, and surgery groups based on treatments received within 18 months from initial diagnosis. Patients in the ATD group received only ATD, while those in RAI and surgery groups could receive ATDs before treatment if RAI or surgery was performed within 18 months of diagnosis. Procedure codes for thyroid surgery and RAI are provided in eTable 2 in Supplement 1.^26^ We set the first 18 months after diagnosis as the observation window to assign exposure types (ATD, RAI, or surgery) because American Thyroid Association (ATA) guidelines suggest considering definitive treatment with RAI or surgery if remission is not achieved with ATDs by that time.^9^ Follow-up began from the index date, which was set at 18 months after the initial hyperthyroidism diagnosis; this landmark approach avoided immortal time bias in our analyses (eFigure 1 and eAppendix 2 in Supplement 1).^27,28^

We excluded patients who had ever taken ATDs, undergone RAI treatment, or received thyroid surgery before hyperthyroidism diagnosis. Patients with a thyroid cancer history or individuals pregnant within 12 months before diagnosis were excluded (eTable 3 in Supplement 1).^29,30^ We also excluded patients who received no treatment or who received both RAI and surgery within 18 months after diagnosis for analyses of primary outcomes. However, patients who had both RAI and surgery were included in hyperthyroidism relapse risk analyses. We excluded patients who withdrew from the National Health Insurance program or died before the index date.

### Study Outcomes and Follow-Up

Primary outcomes were MACE (a composite of AMI, stroke, heart failure, and cardiovascular mortality) and all-cause mortality. Inpatient diagnosis codes for the MACE composite outcome were required to avoid misclassification. The first inpatient MACE diagnosis during the follow-up period was defined as the event date. MACE diagnosis code accuracy has been validated in prior analyses of administrative databases, including NHIRD.^31^ MACE diagnosis codes are summarized in eTable 4 in Supplement 1. The National Register of Deaths was cross-checked to ascertain mortality. Patients who had any MACE outcome or who died from any cause before the index date were excluded from analyses for the corresponding outcome.

Secondary outcomes included individual MACE components and hyperthyroidism relapse. Definitions of relapse differed by treatment group. In the ATD group, relapse was defined as requiring treatment with ATDs, RAI, or surgery after 18 months of ATD therapy. For the RAI group, relapse was defined as continued ATD use 6 months after RAI treatment or requiring another RAI treatment or surgery after the initial RAI treatment. The 6-month observation period was chosen based on ATA guideline recommendations.^9^ Patients in the surgery group who continued to require ATD treatment 3 months after surgery or received either RAI or repeated thyroid surgery any time after initial surgery were considered to have relapsed.

Individuals were followed up from the index date until the date of outcome development, death, insurance withdrawal, or December 31, 2020, whichever came first. Patients were tracked until the specific outcome was observed for each analysis; patients were not censored for the outcome of interest if other outcomes occurred earlier.

### Covariates and Confounders

We considered baseline comorbidities associated with cardiovascular disease to be diagnoses (eTable 5 in Supplement 1) received at least once as an inpatient or twice as an outpatient within 1 year prior to the index date (Table 1). Thyroid cancer was considered separately from other cancers because of the high thyroid cancer rate in hyperthyroidism.^32,33,34^ The Charlson Comorbidity Index was calculated to assess overall comorbidity status.^35^ Medications associated with cardiovascular disease (aspirin, anticoagulants, antihypertensives, antidiabetics, and lipid-lowering agents) were considered baseline medications if prescribed for 30 days or more within 1 year before the index date (Table 1).

**Table 1.** Baseline Characteristics and Comorbidities of Patients Analyzed for Composite Outcome^a^

Baseline characteristic	Patients, No. (%)		SMD^b^	Patients, No. (%)			SMD^b^	Patients, No. (%)		SMD^b^
	ATD (n = 107 044)	RAI (n = 1240)		ATD (n = 107 054)	Surgery (n = 5720)			RAI (n = 1229)	Surgery (n = 5780)	
Age, y										
Mean (SD)	44.0 (13.6)	44.3 (13.5)	0.022	44.1 (13.6)		44.1 (13.8)	0.004	46.7 (13.8)	46.3 (13.6)	0.029
20-54	83 533 (78.0)	980 (79.0)	0.024	83176 (77.7)		4348 (76.0)	0.040	898 (73.0)	4112 (71.1)	0.042
≥55	23 510 (22.0)	260 (21.0)	0.024	23878 (22.3)		1372 (24.0)	0.040	332 (27.0)	1669 (28.9)	0.042
Sex										
Male	29 236 (27.3)	332 (26.7)	0.013	28712 (26.8)		1506 (26.3)	0.011	225 (18.3)	1067 (18.5)	0.004
Female	77 808 (72.7)	909 (73.3)	0.013	78341 (73.2)		4214 (73.7)	0.011	1004 (81.7)	4713 (81.5)	0.004
CCI, mean (SD)	0.3 (0.8)	0.3 (0.8)	0.009	0.3 (0.9)		0.4 (0.9)	0.029	0.7 (1.3)	0.7 (1.3)	0.002
Comorbidity										
Hypertension	13 644 (12.7)	167 (13.5)	0.021	13 995 (13.1)		798 (13.9)	0.026	228 (18.5)	1051 (18.2)	0.009
Coronary artery disease	2172 (2.0)	30 (2.4)	0.025	2207 (2.1)		132 (2.3)	0.016	44 (3.6)	147 (2.5)	0.062
COPD	1951 (1.8)	25 (2.0)	0.014	2007 (1.9)		113 (2.0)	0.007	38 (3.1)	143 (2.5)	0.036
Chronic kidney disease	1080 (1.0)	13 (1.0)	0.001	1112 (1.0)		64 (1.1)	0.008	22 (1.8)	103 (1.8)	0.002
Liver cirrhosis	2593 (2.4)	29 (2.4)	0.005	2598 (2.4)		155 (2.7)	0.018	32 (2.6)	147 (2.5)	0.005
Hyperlipidemia	9841 (9.2)	117 (9.4)	0.008	9991 (9.3)		514 (9.0)	0.012	126 (10.3)	622 (10.8)	0.016
Diabetes	6507 (6.1)	82 (6.6)	0.021	6642 (6.2)		358 (6.3)	0.002	91 (7.4)	442 (7.6)	0.009
Atrial fibrillation	590 (0.6)	7 (0.6)	0.002	578 (0.5)		31 (0.5)	0.001	4 (0.3)	17 (0.3)	0.001
Peripheral vascular disease	192 (0.2)	2 (0.2)^c^	0.003	188 (0.2)		8 (0.1)	0.008	2 (0.1)^c^	9 (0.2)	0.006
Rheumatoid arthritis	185 (0.2)	2 (0.2)^c^	0.006	192 (0.2)		12 (0.2)	0.006	5 (0.4)	14 (0.2)	0.034
Gout	1488 (1.4)	17 (1.4)	0.001	1481 (1.4)		73 (1.3)	0.010	14 (1.1)	76 (1.3)	0.014
Deep vein thrombosis	61 (0.1)	1 (0.1)^c^	0.001	56 (0.1)		1 (<0.1)^c^	0.016	1 (0.1)^c^	3 (<0.1)	0.011
Pulmonary embolism	19 (<0.1)	0 (<0.1)^c^	0.019	19 (<0.1)		1 (<0.1)^c^	0.002	0 (<0.1)^c^	1 (<0.1)^c^	0.017
Osteoporosis	878 (0.8)	9 (0.8)	0.006	886 (0.8)		52 (0.9)	0.009	17 (1.4)	61 (1.0)	0.030
All cancers except thyroid cancer	2294 (2.1)	25 (2.0)	0.011	2294 (2.1)		135 (2.4)	0.015	40 (3.3)	196 (3.4)	0.008
Thyroid cancer	449 (0.4)	5 (0.4)	0.001	567 (0.5)		31 (0.5)	0.001	171 (13.9)	773 (13.4)	0.015
Medication use										
Warfarin	277 (0.3)	5 (0.4)	0.030	270 (0.3)		7 (0.1)	0.031	2 (0.2)^c^	10 (0.2)	0.004
NOAC	301 (0.3)	4 (0.3)	0.005	297 (0.3)		20 (0.4)	0.014	2 (0.2)^c^	13 (0.2)	0.016
Aspirin	2563 (2.4)	39 (3.1)	0.044	2617 (2.4)		145 (2.5)	0.006	33 (2.7)	165 (2.9)	0.011
P2Y12 inhibitor	232 (0.2)	10 (0.8)	0.083	235 (0.2)		18 (0.3)	0.018	6 (0.5)	9 (0.2)	0.054
ARB	4953 (4.6)	54 (4.4)	0.012	5032 (4.7)		300 (5.3)	0.025	70 (5.7)	329 (5.7)	0.002
ACEI	751 (0.7)	15 (1.2)	0.055	781 (0.7)		37 (0.6)	0.010	13 (1.0)	53 (0.9)	0.014
α-Blocker	404 (0.4)	4 (0.3)	0.014	407 (0.4)		23 (0.4)	0.003	3 (0.2)	24 (0.4)	0.033
β-Blocker	45 744 (42.7)	550 (44.3)	0.032	44 740 (41.8)		2343 (41.0)	0.017	343 (27.9)	1569 (27.1)	0.018
Calcium channel blocker	7219 (6.7)	89 (7.2)	0.017	7403 (6.9)		419 (7.3)	0.016	124 (10.1)	553 (9.6)	0.017
Diuretic	1675 (1.6)	23 (1.8)	0.021	1684 (1.6)		96 (1.7)	0.009	23 (1.9)	110 (1.9)	0.003
Hydralazine	26 (<0.1)	0 (<0.1)^c^	0.022	29 (<0.1)		1 (<0.1)^c^	0.005	0 (<0.1)^c^	4 (0.1)	0.038
Statin	6827 (6.4)	86 (6.9)	0.022	6953 (6.5)		369 (6.4)	0.002	92 (7.5)	451 (7.8)	0.011
Nonstatin lipid lowering agent	703 (0.7)	6 (0.5)	0.028	728 (0.7)		39 (0.7)	0.001	12 (1.0)	60 (1.0)	0.007
GLP1-RA	24 (<0.1)	0 (<0.1)^c^	0.021	28 (<0.1)		2 (<0.1)^c^	0.000	0 (<0.1)^c^	4 (0.1)	0.038
SGLT2i	190 (0.2)	2 (0.2)^c^	0.005	196 (0.2)		9 (0.2)	0.007	2 (0.2)^c^	15 (0.3)	0.019
Other antidiabetic agent	5533 (5.2)	63 (5.1)	0.004	5623 (5.2)		298 (5.2)	0.002	63 (5.1)	347 (6.0)	0.039
Insulin	807 (0.8)	10 (0.8)	0.008	793 (0.7)		48 (0.8)	0.011	7 (0.6)	31 (0.5)	0.004

Abbreviations: ACEI, angiotensin-converting enzyme inhibitor; ARB, angiotensin II receptor blocker; ATD, antithyroid drug; CCI, Charlson Comorbidity Index; COPD, chronic obstructive pulmonary disease; GLP1-RA, glucagon-like peptide-1 receptor agonist; NOAC, non–vitamin K antagonist oral anticoagulant; RAI, radioactive iodine; SGLT2i, sodium-glucose cotransporter 2 inhibitor; SMD, standardized mean difference.

^a^ A pseudopopulation was constructed by stabilized inverse probability of treatment weighting for analyses.

^b^ An SMD <0.1 indicates a negligible difference.

^c^ In accordance with the data privacy protection regulation of the Ministry of Health and Welfare Statistics Department, specific numbers cannot be disclosed when there are fewer than 3 events. However, the event number presented is calculated using inverse probability of treatment weighting, reflecting a weighted figure rather than the exact count of events.

### Propensity Score–Based Inverse Probability of Treatment Weighting

For each head-to-head comparison, we calculated propensity scores to estimate the probability of receiving the specific treatment (ATDs, surgery, or RAI) using multivariable logistic regression with covariates from Table 1. Propensity score–based stabilized inverse probability of treatment weighting (IPTW) was then applied to create a pseudopopulation with minimal systematic between-group differences in baseline characteristics.^36^ When calculating weights by IPTW, any weight greater than 10 was deemed excessively large and was lowered to this threshold. A method for calculating stabilized weights was also used. This approach of stabilization and truncation of weights helped to prevent unstable or extreme weights for patients with an extremely low probability of receiving a specific treatment.^37,38,39^ IPTW was performed for each head-to-head comparison dataset to minimize between-group differences; these comparisons included the overall analysis and separate outcome, subgroup, stratified, and sensitivity analyses.

### Statistical Analysis

We used the standardized mean difference, with values less than 0.1 indicating a negligible difference, to assess differences in baseline characteristics.^40^ Cumulative incidence curves were estimated by Kaplan-Meier methods, and differences between curves were determined by log-rank tests. Cox proportional hazards models were used to estimate hazard ratios (HRs) for MACE and all-cause mortality. The proportional hazards assumption was checked, and no evidence of violation was found. Logistic regression models were used to estimate the odds ratio (OR) for hyperthyroidism relapse. All analyses were weighted by IPTW to control for potential confounders. A 2-tailed *P* value < .05 was considered statistically significant. Data management and statistical analyses were performed using R statistical software version 4.1.3 (R Project for Statistical Computing). Data were analyzed from October 2022 through December 2023.

Stratified analyses were performed based on several factors: age (<55 and ≥55 years), sex, health care use (divided into higher [top 50%] and lower [bottom 50%] categories based on clinic visit numbers in the year preceding the index date), and index year (2011-2015 and 2016-2020). We performed a sensitivity analysis, extending the landmark time (index date) to 24 months after hyperthyroidism diagnosis, to determine whether a different landmark time would be associated with a difference in results. To account for the association of incidentally discovered thyroid cancer with treatment decisions and outcomes in hyperthyroidism, we performed a sensitivity analysis excluding patients diagnosed with thyroid cancer after hyperthyroidism diagnosis. Additionally, we performed a sensitivity analysis that used propensity score matching to control confounders.

## Results

### Patient Characteristics

**Figure 1.**
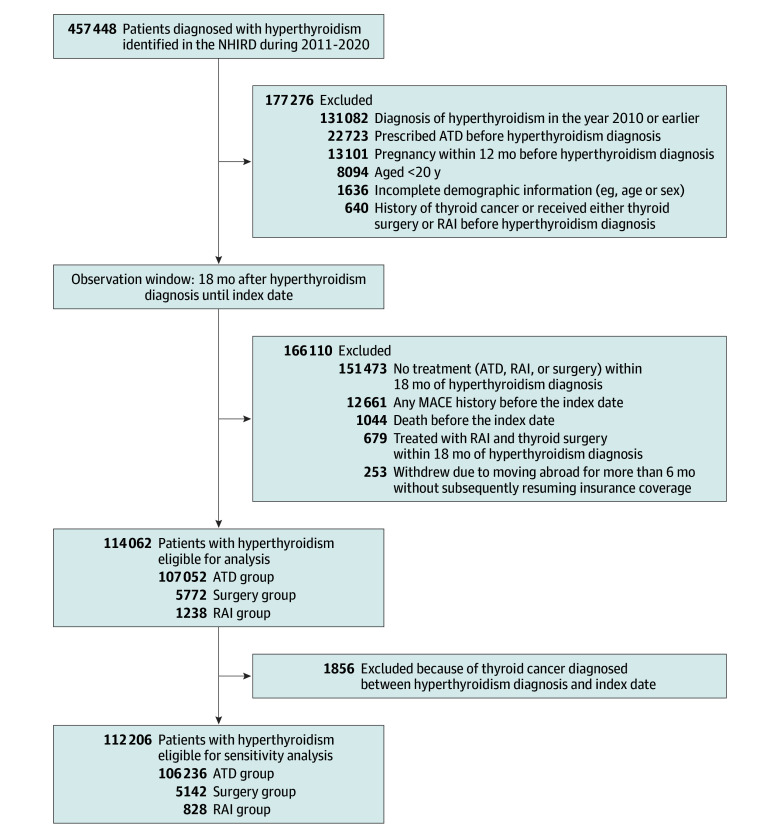
Flowchart of Patient Selection for Composite Outcome Analysis Patient selection for the major adverse cardiovascular events (MACE) composite outcome is shown. ATD indicates antithyroid drug; NHIRD, Taiwan National Health Insurance Research Database; RAI, radioactive iodine.

Figure 1 illustrates patient selection. After applying inclusion and exclusion criteria, a total of 114 062 patients (mean [SD] age, 44.1 [13.6] years; 83 505 female [73.2%]) diagnosed with hyperthyroidism between 2011 and 2020 were included in our main analyses. The mean (SD) follow-up duration was 4.4 (2.5) years. Most patients were treated with ATDs alone (107 052 patients [93.9%]), while 1238 patients (1.1%) received RAI and 5772 patients (5.1%) underwent thyroid surgery. Among patients who underwent surgery, 1019 patients (17.7%) had a total thyroidectomy, 2165 patients (37.5%) had a near-total thyroidectomy, 1479 patients (25.6%) had a lobectomy, 951 patients (16.5%) had a bilateral subtotal thyroidectomy, and 158 patients (2.7%) had a unilateral subtotal thyroidectomy. Unweighted patient baseline characteristics, comorbidities, and cardiovascular medications are presented in eTables 6 and 7 in Supplement 1. After applying IPTW, all variables were balanced between groups, with a standardized mean difference of less than 0.1 for every variable (Table 1; eTable 8 in Supplement 1). After IPTW, there were 107 044 patients receiving ATDs (mean [SD] age, 44.0 [13.6] years; 77 808 females [72.7%]) and 1240 patients receiving RAI (mean [SD] age, 44.3 years; 909 females [73.3%]) in the ATD vs RAI group, 107 054 patients receiving ATDs (mean [SD] age, 44.1 [13.6] years; 78 341 females [73.2%]) and 5720 patients receiving surgery (mean [SD] age, 44.1 [13.8] years; 4214 females [73.7%]) in the ATD vs surgery group, and 1229 patients receiving RAI (mean [SD] age, 46.7 [13.8] years; 1004 females [81.7%]) and 5780 patients receiving surgery (mean [SD] age, 46.3 [13.6] years; 4713 females [81.5%]) in the RAI vs surgery group (Table 1).

### Risks of MACE and All-Cause Mortality

Patients in RAI (HR = 0.45; 95% CI, 0.22-0.93; *P* = .03) and surgery (HR = 0.76; 95% CI, 0.59-0.98, *P* = .04) groups had a lower overall MACE risk compared with patients in the ATD group (Table 2). Patients in the surgery group had significantly lower risks than those in the ATD group for heart failure (HR = 0.33; 95% CI, 0.18-0.59; *P* < .001), cardiovascular mortality (HR = 0.45; 95% CI, 0.26-0.79; *P* = .005), and all-cause mortality (HR = 0.53; 95% CI 0.41-0.68; *P* < .001). Risks for AMI and stroke did not differ significantly between treatment groups.

**Table 2.** Risks of MACE and All-Cause Mortality by Treatment Group^a^

Outcome^b^	Patients, No.	Events, No.	Incidence rate, No./100 000 person-y	HR (95% CI)	*P* value
MACE (composite outcome)					
Comparison 1, ATD vs RAI					
ATD	107 044	1729	374.0	1 [Reference]	.03
RAI	1240	10	170.0	0.45 (0.22-0.93)	
Comparison 2, ATD vs surgery					
ATD	107 054	1742	377.0	1 [Reference]	.04
Surgery	5720	82	290.4	0.76 (0.59-0.98)	
Comparison 3, RAI vs surgery					
RAI	1229	14	246.3	1 [Reference]	.69
Surgery	5780	86	311.8	1.22 (0.45-3.32)	
All-cause mortality					
Comparison 1, ATD vs RAI					
ATD	118 883	2768	542.4	1 [Reference]	.12
RAI	1384	22	361.6	0.67 (0.40-1.11)	
Comparison 2, ATD vs surgery					
ATD	118 862	2786	546.2	1 [Reference]	<.001
Surgery	6345	90	289.4	0.53 (0.41-0.68)	
Comparison 3, RAI vs surgery					
RAI	1378	25	405.6	1 [Reference]	.84
Surgery	6415	116	379.4	0.93 (0.46-1.86)	
AMI					
Comparison 1, ATD vs RAI					
ATD	118 134	285	56.2	1 [Reference]	.51
RAI	1380	2^c^	34.9	0.63 (0.15-2.55)	
Comparison 2, ATD vs surgery					
ATD	118 116	288	56.9	1 [Reference]	.25
Surgery	6320	12	39.8	0.68 (0.35-1.31)	
Comparison 3, RAI vs surgery					
RAI	1374	2^c^	27.9	1 [Reference]	.61
Surgery	6391	13	42.8	1.47 (0.33-6.50)	
Stroke					
Comparison 1, ATD vs RAI					
ATD	111 810	972	201.4	1 [Reference]	.38
RAI	1305	8	141.7	0.71 (0.32-1.54)	
Comparison 2, ATD vs surgery					
ATD	111 809	974	201.9	1 [Reference]	.45
Surgery	5940	67	229.3	1.12 (0.84-1.50)	
Comparison 3, RAI vs surgery					
RAI	1294	13	230.5	1 [Reference]	.97
Surgery	6009	67	234.1	0.98 (0.34-2.84)	
Heart failure					
Comparison 1, ATD vs RAI					
ATD	113 132	780	160.0	1 [Reference]	.25
RAI	1310	5	79.9	0.50 (0.16-1.60)	
Comparison 2, ATD vs surgery					
ATD	113 129	785	161.1	1 [Reference]	<.001
Surgery	6081	16	53.8	0.33 (0.18-0.59)	
Comparison 3, RAI vs surgery					
RAI	1302	3	52.2	1 [Reference]	.99
Surgery	6152	16	55.0	0.99 (0.30-3.32)	
Cardiovascular mortality					
Comparison 1, ATD vs RAI					
ATD	118 883	727	142.4	1 [Reference]	.48
RAI	1384	6	100.3	0.70 (0.27-1.85)	
Comparison 2, ATD vs surgery					
ATD	118 862	734	144.0	1 [Reference]	.005
Surgery	6345	20	65.6	0.45 (0.26-0.79)	
Comparison 3, RAI vs surgery					
RAI	1378	11	184.6	1 [Reference]	.15
Surgery	6415	21	67.6	0.36 (0.09-1.45)	

Abbreviations: AMI, acute myocardial infarction; ATD, antithyroid drug; HR, hazard ratio; MACE, major adverse cardiovascular events; RAI, radioactive iodine.

^a^ A pseudopopulation was constructed by stabilized inverse probability of treatment weighting for analyses.

^b^ In each outcome analysis, patients who had already experienced the corresponding outcome event before the index date were excluded.

^c^ In accordance with the data privacy protection regulation of the Ministry of Health and Welfare Statistics Department, specific numbers cannot be disclosed when there are fewer than 3 events. However, the event number presented is calculated using inverse probability of treatment weighting, reflecting a weighted figure rather than the exact count of events.

**Figure 2.**
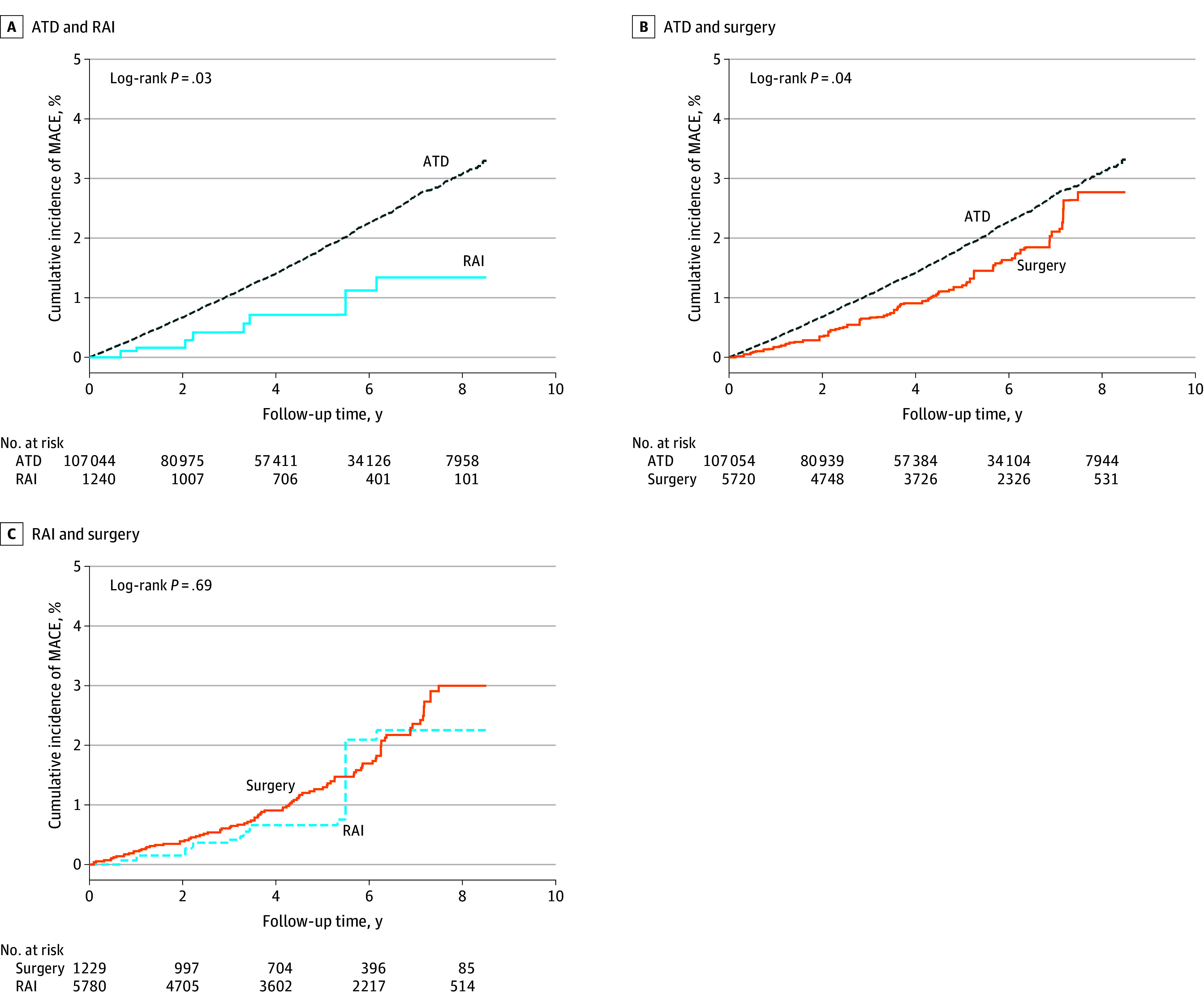
Cumulative Incidence of Composite Outcome by Treatment Group Cumulative incidence of major adverse cardiovascular events (MACE) composite outcome is shown for antithyroid drug (ATD) vs radioactive iodine (RAI) groups (A), ATD vs surgery groups (B), and RAI vs surgery groups (C) after stabilized inverse probability of treatment weighting.

**Figure 3.**
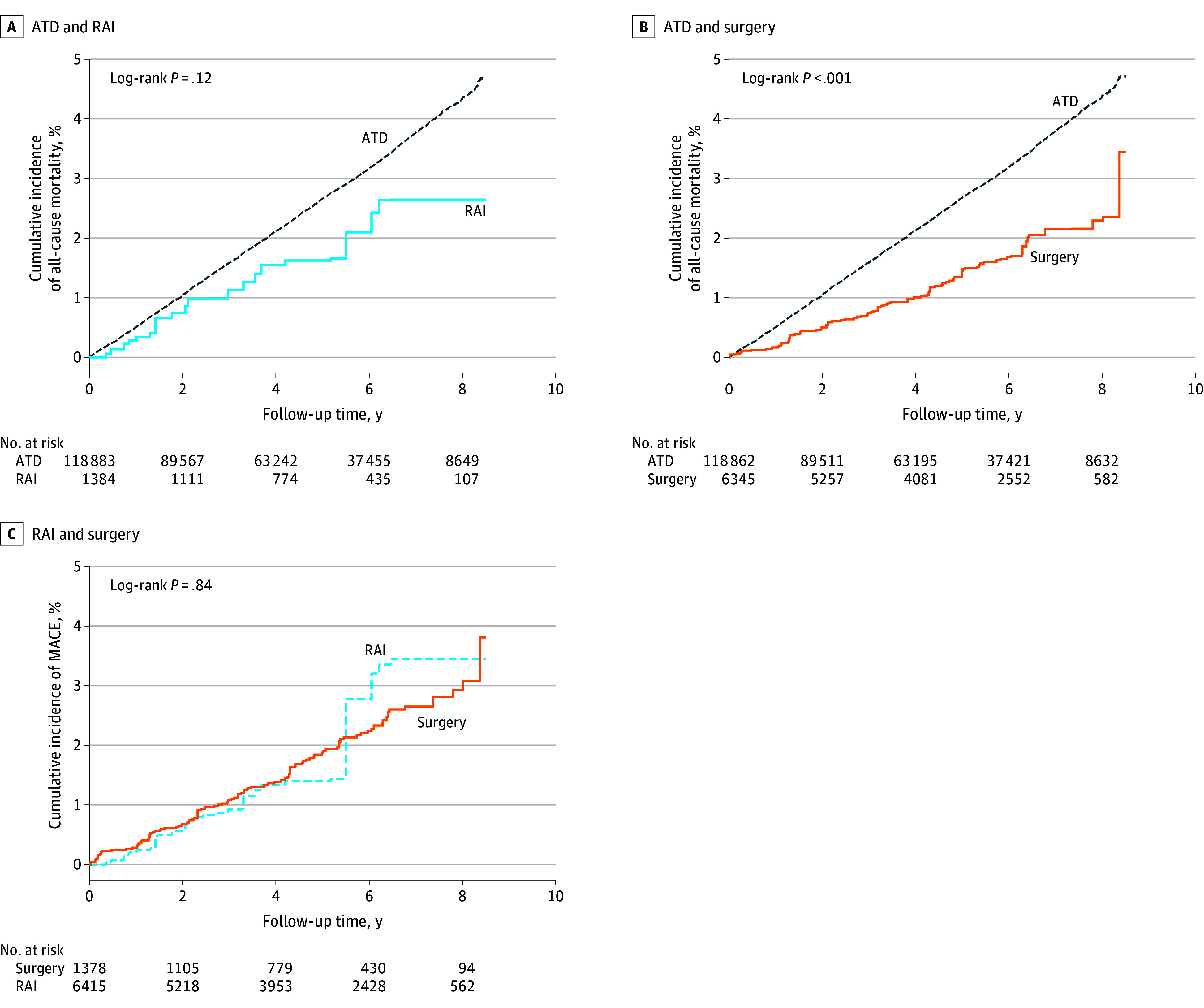
Cumulative Incidence of All-Cause Mortality by Treatment Group Cumulative incidence of all-cause mortality is shown for antithyroid drug (ATD) vs radioactive iodine (RAI) groups (A), ATD vs surgery groups (B), and RAI vs surgery groups (C) after stabilized inverse probability of treatment weighting.

Figure 2 and Figure 3 depict cumulative incidence of MACE and all-cause mortality, respectively, for each comparison set after stabilized IPTW. Crude cumulative incidence curves for MACE and all-cause mortality for each treatment group without stabilized IPTW are shown in eFigure 2 in Supplement 1.

### Risks by Age, Sex, Health Care Use, and Index Year

In stratified analysis, the MACE risk was lower in younger patients (ages 20-54 years) in surgery vs ATD groups (HR = 0.61; 95% CI, 0.40-0.94; *P* = .02). This difference was not observed in patients aged 55 years or older. Patients in both age groups had a lower risk of heart failure (ages 20-54 years: HR = 0.32; 95% CI, 0.13-0.80; *P* = .01; ages ≥55 years: HR = 0.34; 95% CI, 0.16-0.68; *P* = .003) and all-cause mortality (ages 20-54 years: HR = 0.46; 95% CI, 0.26-0.81; *P* = .007; ages ≥55 years: HR = 0.59; 95% CI, 0.45-0.78; *P* < .001) when treated with surgery vs ATDs. There were no significant differences in AMI or stroke regardless of age group (eTable 9 in Supplement 1).

Females treated with surgery compared with ATDs had significantly lower risks of heart failure (HR = 0.33; 95% CI, 0.18-0.61; *P* < .001), cardiovascular mortality (HR = 0.41; 95% CI, 0.19-0.86; *P* = .02), and all-cause mortality (HR = 0.54; 95% CI, 0.40-0.72; *P* < .001). In males, surgery was associated with a reduction in the risk of all-cause mortality compared with ATD treatment (HR = 0.50; 95% CI, 0.31-0.81; *P* = .005) (eTable 10 in Supplement 1).

Patients with lower health care use had significantly lower risks of MACE, heart failure, cardiovascular mortality, and all-cause mortality with surgery compared with ATDs. Higher health care use showed a similar trend, although there were no statistically significant differences in overall MACE and cardiovascular mortality between surgery and ATD groups (eTable 11 in Supplement 1). Stratified analyses by index year period (2011-2015 and 2016-2020) also demonstrated consistent trends across 2 strata (eTable 12 in Supplement 1).

### Hyperthyroidism Relapse

Relapse rates were highest in the ATD group (75 701 of 119 176 patients [63.5%]), followed by RAI (540 of 1413 patients [38.2%]), and surgery (1198 of 6929 patients [17.3%]). Overall, younger individuals (aged 20-54 years) had higher relapse rates than those aged 55 years or older (58 456 of 93 451 patients [62.6%] vs 18 983 of 34 067 patients [55.7%]). The difference in odds of relapse between each group was significant in the overall analysis (RAI vs ATD: OR = 0.43; 95% CI, 0.38-0.48; *P* < .001; surgery vs ATD: OR = 0.08; 95% CI, 0.08-0.09; *P* < .001; surgery vs RAI: OR = 0.45; 95% CI, 0.40-0.51; *P* < .001) (eTable 13 in Supplement 1) and remained similar in analyses stratified by age and sex (eTables 14 and 15 in Supplement 1).

### Results of Sensitivity Analysis

Sensitivity analyses included setting a different landmark time at 24 months after diagnosis (eTable 16 in Supplement 1), excluding patients with incidental thyroid cancer (eTable 17 in Supplement 1), and using propensity score matching (eTable 18 in Supplement 1). All sensitivity analyses yielded results consistent with primary analyses. Although some results did not reach statistical significance, likely due to reduced sample sizes, the overall trends suggesting lower MACE and mortality risks in the surgery and RAI groups remained consistent.

## Discussion

This nationwide cohort study, including a total of 114 062 patients with newly diagnosed hyperthyroidism, found that surgery was associated with a 24% lower MACE risk and 47% lower all-cause mortality risk, while RAI was associated with a 55% lower MACE risk compared with ATDs. Although, to our knowledge, this is the largest study to date comparing risks of different treatments in newly diagnosed hyperthyroidism, regional practice differences restrict the interpretation of our results. In our study, ATDs (93.9%) were the primary first-line treatment for hyperthyroidism. RAI ablation was rarely used (1.1%), similar to patterns reported in Korea,^41^ and this was possibly due to concerns about radiation exposure in densely populated living situations or fears about long-term risks of secondary malignant neoplasms from RAI.^42,43^ Although the proportion of patients undergoing surgery (5.1%) was similar to that in the US (6%)^16^ and slightly higher than in Europe (2.1%)^13^ and the Asia-Pacific region (2%),^14^ total thyroidectomy (17.7% of patients undergoing surgery) was rarely performed in our cohort. Total thyroidectomy is the recommended surgical approach to avoid Graves disease relapse^9^ and is widely performed internationally.^16,17,21^ It is unclear why subtotal thyroidectomies are more common in Taiwan, but this practice may aim to avoid postoperative hypothyroidism or complications.^44^ This unique practice was associated with a high relapse rate (17.3%) among patients undergoing surgery, potentially attenuating outcomes associated with surgery in our outcome analyses.

Our study found significantly lower risks of overall MACE in surgery and RAI groups compared with the ATD group, consistent with findings of a 2021 cohort study^17^ showing that the lowest cumulative incidence of cardiovascular disease was after surgery, followed by RAI, and was highest with ATD treatment. Compared with patients treated with ATDs, patients successfully treated with RAI had a lower risk of MACE in a British linked-record cohort study.^19^ In our study, only heart failure and cardiovascular mortality risks were significantly lower in the surgery group compared with the ATD group among individual MACE outcomes. In Graves disease with preexisting heart failure, total thyroidectomy was associated with improvement in heart failure severity compared with ATDs.^45^ A Swedish study^22^ found that surgery was associated with lower all-cause mortality compared with RAI, primarily due to a lower incidence of cardiovascular mortality. Our study did not find significant differences in individual outcomes of AMI or stroke. Although direct evidence is lacking,^2,46,47^ we speculate that our cohort’s relatively younger age distribution may have been associated with the prevalence of atherosclerotic diseases, contributing to divergent associations with heart failure and other MACE outcomes. Our findings are consistent with those of a previous report^17^ that thyroidectomy for hyperthyroidism was associated with reduced all-cause mortality.

The ATA guideline emphasizes shared decision-making in hyperthyroidism management.^9^ ATDs have become the preferred first-line treatment for hyperthyroidism worldwide,^13,14,15,16^ with proven safety for long-term use.^48,49^ However, it may be time to reassess the role of total thyroidectomy, with emerging evidence showing surgical benefits.^17,18^ Early surgery may be considered in patients with preexisting cardiovascular comorbidities or those at risk of MACE if skilled thyroid surgeons are available to minimize surgical complications.^16^ Alternatively, RAI ablation is an option if the patient is not a surgical candidate or if experienced surgeons are unavailable or based on patient preference.

### Limitations

Several study limitations should be acknowledged. Reliance on *ICD* codes for hyperthyroidism made distinguishing Graves disease from toxic nodular disease challenging, especially when patient-level thyroid function and autoantibody test results were unavailable. Thus, separate evaluation of patients with Graves disease and toxic nodules was not possible. The retrospective nature of the cohort and limited access to detailed patient medical records in the national database restricted exploration of individual factors associated with treatment choices, introducing the potential for indication bias and information bias. Granular clinical information about lifestyle, body mass index, smoking status, and substance use was unavailable. Furthermore, in cases where the sample size was relatively small (as with our RAI group), it was uncertain whether reasons for choosing 1 treatment over another (ie, confounding by indication) were adequately represented in other groups to enable a reliable IPTW. Despite the application of IPTW to control for measured confounders, some unmeasured or uncontrolled confounders at the patient or provider level may still exist. Infrequent use of RAI in Taiwan resulted in disproportionately small case numbers, limiting statistical power for some individual outcome, stratified, and sensitivity analyses. Notably, most point estimates tended to show lower risks with surgery and RAI, although some were not statistically significant and thus did not have associations. Further large-scale studies and meta-analyses are needed to provide additional evidence. Additionally, the extremely low proportion of total thyroidectomy and the high relapse rate in our surgery group likely attenuated results and may impact generalizability.

## Conclusions

In this cohort study of patients with newly diagnosed hyperthyroidism, surgery was associated with lower long-term risks of MACE and all-cause mortality compared with ATDs. RAI was also associated with lower MACE risk compared with ATD use. These findings suggest that surgery or RAI may be better options than long-term ATD treatment in patients with hyperthyroidism who are at risk of MACE. However, these findings should be interpreted with caution owing to the retrospective, observational nature of the study design, which precludes the evaluation of causal relationships. Further large, long-term prospective studies or randomized clinical trials comparing treatment modalities are needed to provide evidence to support patient-centered decision-making.

## Notes

Supplement 1. **eAppendix 1.** Taiwan’s National Health Insurance Research Database **eAppendix 2.** Landmark Analysis and Study Design **eTable 1.** Diagnosis Codes for Hyperthyroidism **eTable 2.** Procedure Codes for Thyroid Surgery and Radioactive Iodine Ablation **eTable 3.** Codes Used to Exclude Pregnant Individuals From the Cohort **eTable 4.** Diagnosis Codes for Major Adverse Cardiovascular Events **eTable 5.** Diagnosis Codes for Baseline Comorbidities **eTable 6.** Baseline Characteristics and Comorbidities of Patients With Hyperthyroidism Analyzed for Composite Outcome Before Inverse Probability of Treatment Weighting **eTable 7.** Baseline Characteristics and Comorbidities of Patients With Hyperthyroidism Analyzed for All-Cause Mortality Before Inverse Probability of Treatment Weighting **eTable 8.** Baseline Characteristics and Comorbidities of Patients With Hyperthyroidism Analyzed for All-Cause Mortality After Inverse Probability of Treatment Weighting **eTable 9.** Risks of Composite Outcome and All-Cause Mortality by Age Among Patients With Hyperthyroidism Treated After Inverse Probability of Treatment Weighting **eTable 10.** Risks of Composite Outcome and All-Cause Mortality by Sex Among Patients With Hyperthyroidism Treated After Inverse Probability of Treatment Weighting **eTable 11.** Risks of Composite Outcome and All-Cause Mortality by Health Care Use Among Patients With Hyperthyroidism Treated After Inverse Probability of Treatment Weighting **eTable 12.** Risks of Composite Outcome and All-Cause Mortality by Index Year Among Patients With Hyperthyroidism Treated After Inverse Probability of Treatment Weighting **eTable 13.** Risks of Hyperthyroidism Relapse in Patients Treated After Inverse Probability of Treatment Weighting **eTable 14.** Risks of Hyperthyroidism Relapse by Age in Patients Treated After Inverse Probability of Treatment Weighting **eTable 15.** Risks of Hyperthyroidism Relapse by Sex in Patients Treated After Inverse Probability of Treatment Weighting **eTable 16.** Sensitivity Analysis: Risks of Composite Outcome and All-Cause Mortality in Patients With Hypothyroidism Treated With Index Date at 24 mo After Diagnosis After Inverse Probability of Treatment Weighting **eTable 17.** Sensitivity Analysis After Excluding Incidental Thyroid Cancer: Risks of Composite Outcome and All-Cause Mortality in Patients With Hypothyroidism Treated With Index Date at 24 mo After Diagnosis After Inverse Probability of Treatment Weighting **eTable 18.** Sensitivity Analysis Using Propensity Score Matching: Risks of Composite Outcome and All-Cause Mortality in Patients With Hypothyroidism Treated With Index Date at 24 mo After Diagnosis After Inverse Probability of Treatment Weighting **eFigure 1.** Illustrated Study Design **eFigure 2.** Crude Cumulative Incidence Curves of Treatments for Composite Outcome and All-Cause Mortality Without Inverse Probability of Treatment Weighting
eReferences.

Supplement 2.
Data Sharing Statement
