# Supplementary material for: MACE and Hyperthyroidism Treated With Medication, Radioactive Iodine, or Thyroidectomy
Source: JAMA Netw Open. 2024 Mar 4;7(3):e240904. doi: 10.1001/jamanetworkopen.2024.0904 (PMC10912964; doi:10.1001/jamanetworkopen.2024.0904)
Supplement: Supplement 1. — eAppendix 1. Taiwan’s National Health Insurance Research Database eAppendix 2. Landmark Analysis and Study Design eTable 1. Diagnosis Codes for Hyperthyroidism eTable 2. Procedure Codes for Thyroid Surgery and Radioactive Iodine Ablation eTable 3. Codes Used to Exclude Pregnant Individuals From the Cohort eTable 4. Diagnosis Codes for Major Adverse Cardiovascular Events eTable 5. Diagnosis Codes for Baseline Comorbidities eTable 6. Baseline Characteristics and Comorbidities of Patients With Hyperthyroidism Analyzed for Composite Outcome Before Inverse Probability of Treatment Weighting eTable 7. Baseline Characteristics and Comorbidities of Patients With Hyperthyroidism Analyzed for All-Cause Mortality Before Inverse Probability of Treatment Weighting eTable 8. Baseline Characteristics and Comorbidities of Patients With Hyperthyroidism Analyzed for All-Cause Mortality After Inverse Probability of Treatment Weighting eTable 9. Risks of Composite Outcome and All-Cause Mortality by Age Among Patients With Hyperthyroidism Treated After Inverse Probability of Treatment Weighting eTable 10. Risks of Composite Outcome and All-Cause Mortality by Sex Among Patients With Hyperthyroidism Treated After Inverse Probability of Treatment Weighting eTable 11. Risks of Composite Outcome and All-Cause Mortality by Health Care Use Among Patients With Hyperthyroidism Treated After Inverse Probability of Treatment Weighting eTable 12. Risks of Composite Outcome and All-Cause Mortality by Index Year Among Patients With Hyperthyroidism Treated After Inverse Probability of Treatment Weighting eTable 13. Risks of Hyperthyroidism Relapse in Patients Treated After Inverse Probability of Treatment Weighting eTable 14. Risks of Hyperthyroidism Relapse by Age in Patients Treated After Inverse Probability of Treatment Weighting eTable 15. Risks of Hyperthyroidism Relapse by Sex in Patients Treated After Inverse Probability of Treatment Weighting eTable 16. Sensitivity Analysis: Risks of Composite [file jamanetwopen-e240904-s001.pdf]

Supplemental Online Content

Peng CCH, Lin YJ, Lee SY, et al. MACE and hyperthyroidism treated with medication, radioactive iodine, or thyroidectomy. *JAMA Netw Open*. 2024;7(3):e240904. doi:10.1001/jamanetworkopen.2024.0904

eAppendix 1. Taiwan's National Health Insurance Research Database

eAppendix 2. Landmark Analysis and Study Design

eTable 1. Diagnosis Codes for Hyperthyroidism

eTable 2. Procedure Codes for Thyroid Surgery and Radioactive Iodine Ablation

eTable 3. Codes Used to Exclude Pregnant Individuals From the Cohort

eTable 4. Diagnosis Codes for Major Adverse Cardiovascular Events

eTable 5. Diagnosis Codes for Baseline Comorbidities

eTable 6. Baseline Characteristics and Comorbidities of Patients With Hyperthyroidism Analyzed for Composite Outcome Before Inverse Probability of Treatment Weighting

eTable 7. Baseline Characteristics and Comorbidities of Patients With Hyperthyroidism Analyzed for All-Cause Mortality Before Inverse Probability of Treatment Weighting

eTable 8. Baseline Characteristics and Comorbidities of Patients With Hyperthyroidism Analyzed for All-Cause Mortality After Inverse Probability of Treatment Weighting

eTable 9. Risks of Composite Outcome and All-Cause Mortality by Age Among Patients With Hyperthyroidism Treated After Inverse Probability of Treatment Weighting

eTable 10. Risks of Composite Outcome and All-Cause Mortality by Sex Among Patients With Hyperthyroidism Treated After Inverse Probability of Treatment Weighting

eTable 11. Risks of Composite Outcome and All-Cause Mortality by Health Care Use Among Patients With Hyperthyroidism Treated After Inverse Probability of Treatment Weighting

eTable 12. Risks of Composite Outcome and All-Cause Mortality by Index Year Among Patients With Hyperthyroidism Treated After Inverse Probability of Treatment Weighting

eTable 13. Risks of Hyperthyroidism Relapse in Patients Treated After Inverse Probability of Treatment Weighting

eTable 14. Risks of Hyperthyroidism Relapse by Age in Patients Treated After Inverse Probability of Treatment Weighting

© 2024 Peng CCH et al. *JAMA Network Open.*

eTable 15. Risks of Hyperthyroidism Relapse by Sex in Patients Treated After Inverse Probability of Treatment Weighting

eTable 16. Sensitivity Analysis: Risks of Composite Outcome and All-Cause Mortality in Patients With Hypothyroidism Treated With Index Date at 24 mo After Diagnosis After Inverse Probability of Treatment Weighting

eTable 17. Sensitivity Analysis After Excluding Incidental Thyroid Cancer: Risks of Composite Outcome and All-Cause Mortality in Patients With Hypothyroidism Treated With Index Date at 24 mo After Diagnosis After Inverse Probability of Treatment Weighting

eTable 18. Sensitivity Analysis Using Propensity Score Matching: Risks of Composite Outcome and All-Cause Mortality in Patients With Hypothyroidism Treated With Index Date at 24 mo After Diagnosis After Inverse Probability of Treatment Weighting

eFigure 1. Illustrated Study Design

eFigure 2. Crude Cumulative Incidence Curves of Treatments for Composite Outcome and All-Cause Mortality Without Inverse Probability of Treatment Weighting

eReferences.

This supplemental material has been provided by the authors to give readers additional information about their work.

© 2024 Peng CCH et al. *JAMA Network Open.*

eNote 1: A brief introduction to Taiwan's National Health Insurance Research Database (NHIRD)

The National Health Insurance program is a mandatory single-payer program administered by the government of Taiwan since 1995 and covers more than 99% of the population of Taiwan. The NHIRD comprises healthcare data from approximately 23.6 million individuals, representing the entire population of Taiwan. The NHIRD includes patient demographic information and medical claims for inpatient, outpatient, and emergency care services. The diagnostic and procedure codes in the NHIRD were derived using the International Classification of Diseases, Ninth Revision, Clinical Modification (ICD-9-CM) codes before 2016 and the International Classification of Diseases, Tenth Revision, Clinical Modification (ICD-10-CM) codes after 2016. The NHIRD is maintained by the Health and Welfare Data Science Center, Ministry of Health and Welfare, Taiwan. For research purposes, anonymized data was made available.

eNote 2: A brief introduction to landmark analysis and our study design

The landmark analysis involves the use of a predetermined landmark time during follow-up to assess subjects who have survived up to that designated point.[1] In studies where there is a delay or waiting period to ascertain a subject's treatment status, immortal time may occur.[2] Therefore, for studies susceptible to immortal time bias, landmark analysis can help prevent such bias by excluding subjects who died or became ineligible before the landmark time.[1] Subjects are then categorized according to their status at the landmark time and the outcome of interest is followed afterwards.

eFigure 1 visually represented our study design. The landmark, which was also the index date, was set at 18 months after diagnosis. Primary outcomes of MACE and all-cause mortality were followed from the index date. Between the date of diagnosis and the index date was the period of treatment assignment. During this timeframe, the timing of when patients initiated each treatment could vary. Those who exclusively received ATD treatment were categorized into the ATD group. Patients in the RAI group and surgery group could receive antithyroid drug before their respective treatments.

eTable 1. Diagnosis codes for hyperthyroidism

	ICD-9-CM	ICD-10-CM
Graves' disease	242.0	E05.0
Toxic nodular disease	242.1, 242.2, 242.3	E05.1, E05.2
Unspecified hyperthyroidism	242.8, 242.9	E05.8, E05.9

eTable 2. Procedure codes for thyroid surgeries and radioactive iodine ablation

Procedure	Code
Unilateral subtotal thyroidectomy	82001C
Bilateral subtotal thyroidectomy	82002C
Unilateral total thyroidectomy	82004B
One side total and another side subtotal thyroidectomy	82015B
Bilateral total thyroidectomy	82016B
Radioactive iodine ablation	26038B

© 2024 Peng CCH et al. *JAMA Network Open.*

eTable 3. Codes used to exclude pregnant women from the cohort

Code types	Pregnancy-related codes
**ICD-9-CM**	630-679
	V22-V24
	V27, V28, V72.42
**ICD-10-CM**	O00-O9A
	Z32-Z39, Z3A
**Procedure codes**	18037B, 18037C, 81024C, 81025C, 81026C, 97001K, 97002A, 97003B, 97004C, 97005D, 81017C, 81034C, 81018C, 81019C, 97931K, 97932A, 97933B, 97934C, 81004C, 81011C, 81028C, 97006K, 97007A, 97008B, 97009C, 97014C, 81029C, 81005B, 81005C, 81006C, 81007C, 81022B, 81008B, 81009C, 81010C, 81030C

eTable 4. Diagnosis codes for major adverse cardiovascular events

	ICD-9-CM	ICD-10-CM
Acute myocardial infarction	410	I21, I22
Stroke	430-437	I60-I67, G45
Heart failure	428	I50
Cardiovascular mortality	390-459	I00-I99

© 2024 Peng CCH et al. *JAMA Network Open.*

eTable 5. Diagnosis codes for baseline comorbidities

Comorbidity	ICD-9-CM	ICD-10-CM
**Hypertension**	401-405	I10-I16
**Coronary artery disease**	410-414	I20-I25
**Congestive heart failure**	428	I50
**Chronic obstructive pulmonary disease**	490-492, 494, 496	J40-J44, J47
**Chronic kidney disease**	582, 583, 585, 586, 588	N03, N04, N05, N18, N19
**Liver cirrhosis**	571	K74
**Hyperlipidemia**	272.0-272.4	E78.0-E78.5
**Cerebrovascular disease**	430-438	I60-I69
**Diabetes mellitus**	250	E10.0, E10.1, E10.9, E11.0, E11.1, E11.9
**Atrial fibrillation**	427.31	I48
**Peripheral vascular disease**	250.7, 443.9, 440.2	E08.5, E10.5, E11.5, E13.5, I73.9, I70.2
**Rheumatoid arthritis**	714.0	M05, M06
**Gout**	274	M10
**Deep vein thrombosis**	453.2, 453.4, 453.5, 453.7, 453.8, 453.9	I82.2, I82.4, I82.5, I82.60, I82.62, I82.89, I82.9
**Pulmonary embolism**	415.1 Pulmonary embolism and infarction	I26 Pulmonary embolism
**Osteoporosis**	733.0, 733.1	M80, M81
**All cancers except thyroid cancer**	140-209, except 193	C00-C96, except C73
**Thyroid cancer**	193	C73

© 2024 Peng CCH et al. *JAMA Network Open.*

eTable 6. Baseline characteristics and comorbidities of patients with hyperthyroidism analyzed for MACE composite outcome before IPTW

Baseline Characteristics	Patients, No. (%)		SMD[a]	Patients, No. (%)		SMD[a]	Patients, No. (%)		SMD[a]
	ATD (n = 107052)	RAI (n = 1238)		ATD (n = 107052)	Surgery (n = 5772)		RAI (n = 1238)	Surgery (n = 5772)	
**Age, mean (SD), y**	44.0 (13.6)	44.9 (13.3)	0.067	44.0 (13.6)	46.6 (13.6)	0.191	44.9 (13.3)	46.6 (13.6)	0.126
**Age group**									
20-54	83582 (78.1)	943 (76.2)	0.045	83582 (78.1)	4058 (70.3)	0.179	943 (76.2)	4058 (70.3)	0.134
≥55	23470 (21.9)	295 (23.8)	0.045	23470 (21.9)	1714 (29.7)	0.179	295 (23.8)	1714 (29.7)	0.134
**Sex**									
Male	29267 (27.3)	299 (24.2)	0.071	29267 (27.3)	991 (17.2)	0.245	299 (24.2)	991 (17.2)	0.173
Female	77785 (72.7)	939 (75.8)	0.071	77785 (72.7)	4781 (82.8)	0.245	939 (75.8)	4781 (82.8)	0.173
**Charlson Comorbidity Index, mean (SD)**	0.3 (0.8)	1.3 (2.0)	0.657	0.3 (0.8)	0.6 (1.1)	0.312	1.3 (2.0)	0.6 (1.1)	0.434
**Comorbidities, %**									
Hypertension	13600 (12.7)	186 (15.0)	0.067	13600 (12.7)	1087 (18.8)	0.169	186 (15.0)	1087 (18.8)	0.102
Coronary artery disease	2176 (2.0)	27 (2.2)	0.010	2176 (2.0)	149 (2.6)	0.037	27 (2.2)	149 (2.6)	0.026
Chronic obstructive pulmonary disease	1959 (1.8)	15 (1.2)	0.051	1959 (1.8)	157 (2.7)	0.060	15 (1.2)	157 (2.7)	0.109
Chronic kidney disease	1062 (1.0)	24 (1.9)	0.079	1062 (1.0)	100 (1.7)	0.064	24 (1.9)	100 (1.7)	0.015
Liver cirrhosis	2585 (2.4)	31 (2.5)	0.006	2585 (2.4)	147 (2.5)	0.008	31 (2.5)	147 (2.5)	0.003
Hyperlipidemia	9840 (9.2)	101 (8.2)	0.037	9840 (9.2)	661 (11.5)	0.074	101 (8.2)	661 (11.5)	0.111
Diabetes mellitus	6515 (6.1)	65 (5.3)	0.036	6515 (6.1)	469 (8.1)	0.079	65 (5.3)	469 (8.1)	0.115
Atrial fibrillation	593 (0.6)	5 (0.4)	0.022	593 (0.6)	16 (0.3)	0.043	5 (0.4)	16 (0.3)	0.022
Peripheral vascular disease	191 (0.2)	4 (0.3)	0.029	191 (0.2)	8 (0.1)	0.010	4 (0.3)	8 (0.1)	0.038
Rheumatoid arthritis	186 (0.2)	1 (0.1)	0.026	186 (0.2)	16 (0.3)	0.022	1 (0.1)	16 (0.3)	0.046
Gout	1481 (1.4)	17 (1.4)	0.001	1481 (1.4)	76 (1.3)	0.006	17 (1.4)	76 (1.3)	0.005
Deep vein thrombosis	58 (0.1)	4 (0.3)	0.062	58 (0.1)	1 (0.0)	0.019	4 (0.3)	1 (0.0)	0.074
Pulmonary embolism	19 (0.0)	0 (0.0)	0.019	19 (0.0)	1 (0.0)	<0.001	0 (0.0)	1 (0.0)	0.019
Osteoporosis	880 (0.8)	13 (1.1)	0.024	880 (0.8)	60 (1.0)	0.023	13 (1.1)	60 (1.0)	0.001
All cancers except thyroid cancer	2240 (2.1)	100 (8.1)	0.275	2240 (2.1)	151 (2.6)	0.035	100 (8.1)	151 (2.6)	0.245
Thyroid cancer	69 (0.1)	394 (31.8)	0.963	69 (0.1)	529 (9.2)	0.444	394 (31.8)	529 (9.2)	0.585

© 2024 Peng CCH et al. *JAMA Network Open.*

Baseline Characteristics	Patients, No. (%)		SMD[a]	Patients, No. (%)		SMD[a]	Patients, No. (%)		SMD[a]
	ATD (n =107052)	RAI (n = 1238)		ATD (n = 107052)	Surgery (n = 5772)		RAI (n = 1238)	Surgery (n = 5772)	
**Medication use**									
Warfarin	277(0.3)	4 (0.3)	0.012	277(0.3)	9 (0.2)	0.023	4 (0.3)	9 (0.2)	0.034
NOAC	303(0.3)	4 (0.3)	0.007	303(0.3)	12 (0.2)	0.015	4 (0.3)	12 (0.2)	0.022
Aspirin	2566 (2.4)	29 (2.3)	0.004	2566 (2.4)	173 (3.0)	0.037	29 (2.3)	173 (3.0)	0.041
P2Y12 inhibitor	233 (5.9)	1 (0.1)	0.347	233 (5.9)	10 (0.2)	0.010	1 (0.1)	10 (0.2)	0.026
ARB	4945 (4.6)	64 (5.2)	0.026	4945 (4.6)	335 (5.8)	0.053	64 (5.2)	335 (5.8)	0.028
ACEi	753 (0.7)	5 (0.4)	0.040	753 (0.7)	59 (1.0)	0.034	5 (0.4)	59 (1.0)	0.074
α-blockers	401 (0.4)	3 (0.2)	0.024	401 (0.4)	27 (0.5)	0.014	3 (0.2)	27 (0.5)	0.038
β-blockers	45762 (42.7)	470 (38.0)	0.098	45762 (42.7)	1395 (24.2)	0.402	470 (38.0)	1395 (24.2)	0.30‡
Calcium channel blockers	7171 (6.7)	98 (7.9)	0.047	7171 (6.7)	573 (9.9)	0.117	98 (7.9)	573 (9.9)	0.071
Diuretics	1659 (1.5)	28 (2.3)	0.052	1659 (1.5)	107 (1.9)	0.024	28 (2.3)	107 (1.9)	0.029
Hydralazine	26 (0.0)	0 (0.0)	0.022	26 (0.0)	5 (0.1)	0.026	0 (0.0)	5 (0.1)	0.042
Statins	6841 (6.4)	60 (4.8)	0.067	6841 (6.4)	489 (8.5)	0.079	60 (4.8)	489 (8.5)	0.146
Non-statin lipid lowering agents	707 (0.7)	9 (0.7)	0.008	707 (0.7)	65 (1.1)	0.050	9 (0.7)	65 (1.1)	0.042
GLP1-RA	24 (0.0)	0 (0.0)	0.021	24 (0.0)	5 (0.1)	0.028	0 (0.0)	5 (0.1)	0.042
SGLT2i	191 (0.2)	2 (0.2)	0.004	191 (0.2)	16 (0.3)	0.021	2 (0.2)	16 (0.3)	0.025
Other anti-diabetic agents	5549 (5.2)	53 (4.3)	0.043	5549 (5.2)	370 (6.4)	0.053	53 (4.3)	370 (6.4)	0.095
Insulin	810 (0.8)	8 (0.6)	0.013	810 (0.8)	28 (0.5)	0.035	8 (0.6)	28 (0.5)	0.021

MACE, major adverse cardiovascular events; ATD, anti-thyroid drug; RAI, radioactive iodine; IPTW, inverse probability of treatment weighting; SMD, standardized mean difference; NOAC, Non-Vitamin K antagonist oral anticoagulants; ARB, Angiotensin II receptor blockers; ACEi, Angiotensin-converting enzyme inhibitors; GLP1-RA, Glucagon-like peptide-1 receptor agonists; SGLT2i, Sodium-glucose cotransporter-2 inhibitors

[a] A standardized mean difference of < 0.1 indicates a negligible difference.

© 2024 Peng CCH et al. *JAMA Network Open.*

eTable 7. Baseline characteristics and comorbidities of patients with hyperthyroidism analyzed for all-cause mortality before IPTW

Baseline Characteristics	Patients, No. (%)			Patients, No. (%)			Patients, No. (%)		
	ATD (n = 118877)	RAI (n = 1387)	SMD[a]	ATD (n = 118877)	Surgery (n = 6400)	SMD[a]	RAI (n = 1387)	Surgery (n = 6400)	SMD[a]
**Age, mean (SD), y**	45.7 (14.6)	46.4 (14.3)	0.048	45.7 (14.6)	48.1 (14.3)	0.166	46.4 (14.3)	48.1 (14.3)	0.119
**Age group**									
20-54	87621 (73.7)	1005 (72.5)	0.027	87621 (73.7)	4219 (65.9)	0.171	1005 (72.5)	4219 (65.9)	0.143
≥55	31256 (26.3)	382 (27.5)	0.027	31256 (26.3)	2181 (34.1)	0.171	382 (27.5)	2181 (34.1)	0.143
**Sex**									
Male	33106 (27.8)	335 (24.2)	0.082	33106 (27.8)	1104 (17.2)	0.256	335 (24.2)	1104 (17.2)	0.173
Female	85771 (72.2)	1052 (75.8)	0.082	85771 (72.2)	5296 (82.8)	0.256	1052 (75.8)	5296 (82.8)	0.173
**Charlson Comorbidity Index, mean (SD)**	0.4 (1.0)	1.3 (2.0)	0.569	0.4 (1.0)	0.7 (1.2)	0.272	1.3 (2.0)	0.7 (1.2)	0.364
**Comorbidities**									
Hypertension	18720 (15.7)	247 (17.8)	0.055	18720 (15.7)	1440 (22.5)	0.172	247 (17.8)	1440 (22.5)	0.117
Coronary artery disease	4148 (3.5)	47 (3.4)	0.006	4148 (3.5)	240 (3.8)	0.014	47 (3.4)	240 (3.8)	0.019
Congestive heart failure	2231 (1.9)	31 (2.2)	0.025	2231 (1.9)	72 (1.1)	0.062	31 (2.2)	72 (1.1)	0.086
Chronic obstructive pulmonary disease	2884 (2.4)	26 (1.9)	0.038	2884 (2.4)	216 (3.4)	0.057	26 (1.9)	216 (3.4)	0.094
Chronic kidney disease	1848 (1.6)	31 (2.2)	0.050	1848 (1.6)	140 (2.2)	0.047	31 (2.2)	140 (2.2)	0.003
Liver cirrhosis	2974 (2.5)	43 (3.1)	0.036	2974 (2.5)	170 (2.7)	0.010	43 (3.1)	170 (2.7)	0.027
Hyperlipidemia	12307 (10.4)	128 (9.2)	0.038	12307 (10.4)	839 (13.1)	0.086	128 (9.2)	839 (13.1)	0.123
Cerebrovascular disease	2268 (1.9)	19 (1.4)	0.042	2268 (1.9)	133 (2.1)	0.012	19 (1.4)	133 (2.1)	0.054
Diabetes mellitus	8771 (7.4)	82 (5.9)	0.059	8771 (7.4)	602 (9.4)	0.073	82 (5.9)	602 (9.4)	0.132
Atrial fibrillation	1517 (1.3)	14 (1.0)	0.025	1517 (1.3)	37 (0.6)	0.073	14 (1.0)	37 (0.6)	0.049
Peripheral vascular disease	355 (0.3)	5 (0.4)	0.011	355 (0.3)	12 (0.2)	0.023	5 (0.4)	12 (0.2)	0.033
Rheumatoid arthritis	237 (0.2)	1 (0.1)	0.035	237 (0.2)	20 (0.3)	0.022	1 (0.1)	20 (0.3)	0.055
Gout	1891 (1.6)	21 (1.5)	0.006	1891 (1.6)	100 (1.6)	0.002	21 (1.5)	100 (1.6)	0.004
Deep vein thrombosis	104 (0.1)	5 (0.4)	0.058	104 (0.1)	2 (0.0)	0.023	5 (0.4)	2 (0.0)	0.075
Pulmonary embolism	36 (0.0)	0 (0.0)	0.025	36 (0.0)	1 (0.0)	0.010	0 (0.0)	1 (0.0)	0.018
Osteoporosis	1359 (1.1)	16 (1.2)	0.001	1359 (1.1)	86 (1.3)	0.018	16 (1.2)	86 (1.3)	0.017
All cancers except thyroid cancer	2804 (2.4)	107 (7.7)	0.247	2804 (2.4)	179 (2.8)	0.028	107 (7.7)	179 (2.8)	0.222

© 2024 Peng CCH et al. *JAMA Network Open.*

Baseline Characteristics	Patients, No. (%)		SMD[a]	Patients, No. (%)		SMD[a]	Patients, No. (%)		SMD[a]
	ATD (n = 118877)	RAI (n = 1387)		ATD (n = 118877)	Surgery (n = 6400)		RAI (n = 1387)	Surgery (n = 6400)	
**Comorbidities**									
Thyroid cancer	76 (0.1)	423 (30.5)	0.933	76 (0.1)	610 (9.5)	0.454	423 (30.5)	610 (9.5)	0.543
**Medication use**									
Warfarin	879 (0.7)	10 (0.7)	0.002	879 (0.7)	21 (0.3)	0.056	10 (0.7)	21 (0.3)	0.054
NOAC	1291 (1.1)	16 (1.2)	0.006	1291 (1.1)	31 (0.5)	0.068	16 (1.2)	31 (0.5)	0.074
Aspirin	5240 (4.4)	59 (4.3)	0.008	5240 (4.4)	293 (4.6)	0.008	59 (4.3)	293 (4.6)	0.016
P2Y12 inhibitor	912 (0.8)	5 (0.4)	0.054	912 (0.8)	38 (0.6)	0.021	5 (0.4)	38 (0.6)	0.034
ARB	7563 (6.4)	94 (6.8)	0.017	7563 (6.4)	478 (7.5)	0.044	94 (6.8)	478 (7.5)	0.027
ACEi	1300 (1.1)	13 (0.9)	0.016	1300 (1.1)	79 (1.2)	0.013	13 (0.9)	79 (1.2)	0.029
α-blockers	667 (0.6)	7 (0.5)	0.008	667 (0.6)	36 (0.6)	0.000	7 (0.5)	36 (0.6)	0.008
β-blockers	52392 (44.1)	560 (40.4)	0.075	52392 (44.1)	1669 (26.1)	0.384	560 (40.4)	1669 (26.1)	0.307
Calcium channel blockers	10226 (8.6)	141 (10.2)	0.054	10226 (8.6)	759 (11.9)	0.108	141 (10.2)	759 (11.9)	0.054
Diuretics	4028 (3.4)	62 (4.5)	0.056	4028 (3.4)	190 (3.0)	0.024	62 (4.5)	190 (3.0)	0.079
Hydralazine	89 (0.1)	1 (0.1)	0.001	89 (0.1)	10 (0.2)	0.024	1 (0.1)	10 (0.2)	0.025
Statins	9541 (8.0)	79 (5.7)	0.092	9541 (8.0)	653 (10.2)	0.076	79 (5.7)	653 (10.2)	0.167
Non-statin lipid lowering agents	956 (0.8)	10 (0.7)	0.010	956 (0.8)	77 (1.2)	0.040	10 (0.7)	77 (1.2)	0.049
GLP1-RA	38 (0.0)	0 (0.0)	0.025	38 (0.0)	6 (0.1)	0.025	0 (0.0)	6 (0.1)	0.043
SGLT2i	280 (0.2)	4 (0.3)	0.010	280 (0.2)	18 (0.3)	0.009	4 (0.3)	18 (0.3)	0.001
Other anti-diabetic agents	7660 (6.4)	69 (5.0)	0.063	7660 (6.4)	488 (7.6)	0.046	69 (5.0)	488 (7.6)	0.109
Insulin	1188 (1.0)	10 (0.7)	0.030	1188 (1.0)	43 (0.7)	0.036	10 (0.7)	43 (0.7)	0.006

ATD, anti-thyroid drug; RAI, radioactive iodine; IPTW, inverse probability of treatment weighting; SMD, standardized mean difference; NOAC, Non-Vitamin K antagonist oral anticoagulants; ARB, Angiotensin II receptor blockers; ACEi, Angiotensin-converting enzyme inhibitors; GLP1-RA, Glucagon-like peptide-1 receptor agonists; SGLT2i, Sodium-glucose cotransporter-2 inhibitors

[a] A standardized mean difference of < 0.1 indicates a negligible difference.

© 2024 Peng CCH et al. *JAMA Network Open.*

eTable 8. Baseline characteristics and comorbidities of patients with hyperthyroidism analyzed for all-cause mortality after IPTW[a]

Baseline Characteristics	Patients, No. (%)			Patients, No. (%)			Patients, No. (%)		
	ATD (n = 118883)	RAI (n = 1384)	SMD[b]	ATD (n = 118862)	Surgery (n = 6345)	SMD[b]	RAI (n = 1378)	Surgery (n = 6415)	SMD[b]
**Age, mean (SD), y**	45.7 (14.6)	45.9 (14.7)	0.018	45.8 (14.7)	46.0 (14.5)	0.013	48.5 (14.9)	47.8(14.3)	0.046
**Age group**									
20-54	87586 (73.7)	1029 (74.4)	0.015	87222 (73.4)	4484 (70.7)	0.060	933 (67.7)	4268 (66.5)	0.025
≥55	31297 (26.3)	355(25.6)	0.015	31640 (26.6)	1861 (29.3)	0.060	445 (32.3)	2147 (33.5)	0.025
**Sex**									
Male	33060 (27.8)	385 (27.8)	0.001	32459 (27.3)	1684 (26.5)	0.017	256 (18.6)	1187 (18.5)	0.002
Female	85822 (72.2)	1000 (72.2)	0.001	86403 (72.7)	4661 (73.5)	0.017	1122 (81.4)	5228 (81.5)	0.002
**Charlson Comorbidity Index, mean (SD)**	0.5 (1.0)	0.5 (0.9)	0.001	0.5 (1.0)	0.5 (1.0)	0.034	0.8 (1.4)	0.8 (1.4)	0.007
**Comorbidities**									
Hypertension	18771 (15.8)	226 (16.3)	0.015	19167 (16.1)	1089 (17.2)	0.028	310 (22.5)	1396 (21.8)	0.018
Coronary artery disease	4142 (3.5)	49 (3.5)	0.003	4158 (3.5)	247 (3.9)	0.021	64 (4.6)	239 (3.7)	0.044
Congestive heart failure	2248 (1.9)	32 (2.3)	0.029	2189 (1.8)	139 (2.2)	0.025	21 (1.5)	95 (1.5)	0.003
Chronic obstructive pulmonary disease	2882 (2.4)	37 (2.7)	0.018	2942 (2.5)	180 (2.8)	0.022	53 (3.8)	201 (3.1)	0.037
Chronic kidney disease	1868 (1.6)	22 (1.6)	0.003	1898 (1.6)	105 (1.7)	0.004	30 (2.2)	143 (2.2)	0.004
Liver cirrhosis	2989 (2.5)	35 (2.5)	0.000	2987 (2.5)	172 (2.7)	0.013	42 (3.1)	176 (2.7)	0.019
Hyperlipidemia	12309 (10.4)	147 (10.6)	0.010	12486 (10.5)	660 (10.4)	0.004	171 (12.4)	792 (12.4)	0.002
Cerebrovascular disease	2258 (1.9)	27 (1.9)	0.003	2264 (1.9)	137 (2.2)	0.018	38 (2.8)	126 (2.0)	0.052
Diabetes mellitus	8759 (7.4)	110 (7.9)	0.021	8904 (7.5)	499 (7.9)	0.014	125 (9.1)	565 (8.8)	0.010
Atrial fibrillation	1516 (1.3)	16 (1.2)	0.008	1479 (1.2)	93 (1.5)	0.019	10 (0.7)	42 (0.7)	0.007
Peripheral vascular disease	355 (0.3)	4 (0.3)	0.002	347 (0.3)	14 (0.2)	0.015	2 (0.2)[c]	13 (0.2)	0.007
Rheumatoid arthritis	235 (0.2)	3 (0.2)	0.008	243 (0.2)	15 (0.2)	0.007	6 (0.4)	17 (0.3)	0.027
Gout	1896 (1.6)	22 (1.6)	0.002	1891 (1.6)	90 (1.4)	0.014	21 (1.5)	103 (1.6)	0.009
Deep vein thrombosis	108 (0.1)	1 (0.1)[c]	0.001	101 (0.1)	3 (0.1)	0.013	1 (0.1)[c]	5 (0.1)	0.003
Pulmonary embolism	36 (0.0)	0 (0.0)[c]	0.024	35 (0.0)	3 (0.0)	0.008	0 (0.0)[c]	1 (0.0)[c]	0.016
Osteoporosis	1354 (1.1)	13 (0.9)	0.020	1359 (1.1)	76 (1.2)	0.005	23 (1.7)	85 (1.3)	0.032
All cancers except thyroid cancer	2858 (2.4)	31 (2.2)	0.013	2855 (2.4)	165 (2.6)	0.012	48 (3.5)	228 (3.6)	0.006

© 2024 Peng CCH et al. *JAMA Network Open.*

Baseline Characteristics	Patients, No. (%) ATD (n = 118883)	Patients, No. (%) RAI (n = 1384)	SMD[b]	Patients, No. (%) ATD (n = 118862)	Patients, No. (%) Surgery (n = 6345)	SMD[b]	Patients, No. (%) RAI (n = 1378)	Patients, No. (%) Surgery (n = 6415)	SMD[b]
**Comorbidities**									
Thyroid cancer	499 (0.4)	6 (0.4)	0.001	634 (0.5)	35 (0.6)	0.003	194 (14.1)	870 (13.6)	0.014
**Medication use**									
Warfarin	877 (0.7)	9 (0.6)	0.011	852 (0.7)	50 (0.8)	0.007	6 (0.4)	27 (0.4)	0.003
NOAC	1288 (1.1)	16 (1.2)	0.008	1251 (1.1)	79 (1.2)	0.018	9 (0.6)	38 (0.6)	0.003
Aspirin	5241 (4.4)	65 (4.7)	0.014	5259 (4.4)	303 (4.8)	0.017	62 (4.5)	293 (4.6)	0.004
P2Y12 inhibitor	908 (0.8)	13 (1.0)	0.022	906 (0.8)	53 (0.8)	0.009	8 (0.6)	36 (0.6)	0.001
ARB	7589 (6.4)	89 (6.4)	0.001	7647 (6.4)	452 (7.1)	0.028	110 (8.0)	479 (7.5)	0.020
ACEi	1300 (1.1)	17 (1.2)	0.013	1319 (1.1)	67 (1.1)	0.005	12 (0.9)	76 (1.2)	0.029
α-blockers	674 (0.6)	11 (0.8)	0.031	666 (0.6)	38 (0.6)	0.006	11 (0.8)	36 (0.6)	0.033
β-blockers	52394 (44.1)	624 (45.1)	0.021	51279 (43.1)	2682 (42.3)	0.018	412 (29.9)	1873 (29.2)	0.015
Calcium channel blockers	10288 (8.7)	126 (9.1)	0.015	10472 (8.8)	601 (9.5)	0.023	171 (12.4)	749 (11.7)	0.023
Diuretics	4066 (3.4)	53 (3.8)	0.023	4022 (3.4)	238 (3.7)	0.020	49 (3.5)	216 (3.4)	0.009
Hydralazine	89 (0.1)	2 (0.1)[c]	0.020	94 (0.1)	4 (0.1)	0.004	3 (0.3)	9 (0.1)	0.025
Statins	9514 (8.0)	112 (8.1)	0.004	9652 (8.1)	512 (8.1)	0.002	128 (9.3)	603 (9.4)	0.003
Non-statin lipid lowering agents	950 (0.8)	9 (0.7)	0.017	976 (0.8)	51 (0.8)	0.002	17 (1.2)	70 (1.1)	0.011
GLP1-RA	38 (0.0)	0 (0.0)[c]	0.025	42 (0.0)	2 (0.0)[c]	0.001	0 (0.0)[c]	5 (0.1)	0.039
SGLT2i	287 (0.2)	4 (0.3)	0.003	284 (0.2)	13 (0.2)	0.007	3 (0.2)	19 (0.3)	0.013
Other anti-diabetic agents	7649 (6.4)	93 (6.7)	0.011	7742 (6.5)	433 (6.8)	0.012	92 (6.7)	458 (7.1)	0.017
Insulin	1184 (1.0)	14 (1.0)	0.001	1169 (1.0)	78 (1.2)	0.024	11 (0.8)	46 (0.7)	0.007

ATD, anti-thyroid drug; RAI, radioactive iodine; IPTW, inverse probability of treatment weighting; SMD, standardized mean difference; NOAC, Non-Vitamin K antagonist oral anticoagulants; ARB, Angiotensin II receptor blockers; ACEi, Angiotensin-converting enzyme inhibitors; GLP1-RA, Glucagon-like peptide-1 receptor agonists; SGLT2i, Sodium-glucose cotransporter-2 inhibitors

[a] A pseudopopulation constructed by stabilized IPTW for analyses.

[b] A standardized mean difference of < 0.1 indicates a negligible difference.

[c] In accordance with the data privacy protection regulation of the Ministry of Health and Welfare's Statistics Department, specific numbers cannot be disclosed when there are fewer than 3 events. However, the event number presented is calculated using inverse probability of treatment weighting, reflecting a weighted figure rather than the exact count of events.

© 2024 Peng CCH et al. *JAMA Network Open.*

eTable 9. Risks of MACE and all-cause mortality stratified by age among patients with hyperthyroidism treated with ATD, RAI or surgery after IPTW[a]

Outcome[b]	Age group, yr	Treatment	Patients, n	Event number	Incidence Rate[c]	HR (95% CI)	*p*-value
**MACE (composite outcome)**	20-54	ATD	83,576	749	204.4	1.00 (reference)	0.07
		RAI	940	3[d]	58.1	0.28 (0.07-1.12)	
		ATD	83,543	746	203.8	1.00 (reference)	0.02
		Surgery	4,042	26	127.0	0.61 (0.40-0.94)	
		RAI	929	3[d]	70.7	1.00 (reference)	0.21
		Surgery	4,063	28	138.6	1.86 (0.71-4.87)	
	≥55	ATD	23,461	979	1021.1	1.00 (reference)	0.31
		RAI	291	8	669.5	0.65 (0.29-1.48)	
		ATD	23,486	976	1017.1	1.00 (reference)	0.42
		Surgery	1,688	68	903.0	0.88 (0.65-1.19)	
		RAI	291	9	701.6	1.00 (reference)	0.86
		Surgery	1,715	58	777.8	1.10 (0.38-3.21)	
**All-cause mortality**	20-54	ATD	87,617	647	168.3	1.00 (reference)	0.27
		RAI	1,003	5	98.8	0.59 (0.23-1.52)	
		ATD	87,595	644	167.6	1.00 (reference)	0.007
		Surgery	4,196	17	78.3	0.46 (0.26-0.81)	
		RAI	991	3[d]	67.8	1.00 (reference)	0.29
		Surgery	4,220	23	110.3	1.63 (0.65-4.08)	
	≥55	ATD	31,255	2,120	1681.8	1.00 (reference)	0.99
		RAI	384	27	1691.5	1.01 (0.46-2.22)	
		ATD	31,257	2,097	1662.6	1.00 (reference)	<0.001
		Surgery	2,161	94	987.7	0.59 (0.45-0.78)	
		RAI	376	23	1464.8	1.00 (reference)	0.28
		Surgery	2,189	93	977.8	0.67 (0.32-1.38)	

© 2024 Peng CCH et al. *JAMA Network Open.*

Outcome[b]	Age group, yr	Treatment	Patients, n	Event number	Incidence Rate[c]	HR (95% CI)	*p*-value
**Acute myocardial infarction**	20-54	ATD	87,373	119	30.9	1.00 (reference)	0.87
		RAI	999	1[d]	26.0	0.84 (0.12-6.03)	
		ATD	87,351	117	30.7	1.00 (reference)	0.31
		Surgery	4,192	4	17.7	0.55 (0.17-1.74)	
		RAI	988	1[d]	17.1	1.00 (reference)	0.86
		Surgery	4,218	5	22.3	1.22 (0.14-10.82)	
	≥55	ATD	30,752	165	133.0	1.00 (reference)	0.98
		RAI	381	2[d]	128.3	0.98 (0.14-7.04)	
		ATD	30,753	169	135.7	1.00 (reference)	0.29
		Surgery	2,142	9	90.4	0.66 (0.31-1.42)	
		RAI	375	1[d]	86.9	1.00 (reference)	0.97
		Surgery	2,168	8	88.9	0.97 (0.12-7.49)	
**Stroke**	20-54	ATD	85,476	374	99.7	1.00 (reference)	0.20
		RAI	973	1[d]	28.8	0.29 (0.04-1.94)	
		ATD	85,449	372	99.3	1.00 (reference)	0.48
		Surgery	4,096	18	84.8	0.83 (0.50-1.39)	
		RAI	961	2[d]	50.8	1.00 (reference)	0.34
		Surgery	4,120	19	90.8	1.70 (0.57-5.05)	
	≥55	ATD	26,329	597	555.8	1.00 (reference)	0.90
		RAI	336	8	580.3	1.05 (0.45-2.45)	
		ATD	26,339	593	551.2	1.00 (reference)	0.15
		Surgery	1,856	58	712.4	1.29 (0.91-1.82)	
		RAI	331	9	671.2	1.00 (reference)	0.81
		Surgery	1,883	49	599.7	0.88 (0.30-2.59)	

© 2024 Peng CCH et al. *JAMA Network Open.*

Outcome[b]	Age group, yr	Treatment	Patients, n	Event number	Incidence Rate[c]	HR (95% CI)	*p*-value
Heart failure	20-54	ATD	85,731	300	79.8	1.00 (reference)	0.28
		RAI	968	1[d]	27.3	0.34 (0.05-2.43)	
		ATD	85,700	299	79.6	1.00 (reference)	0.01
		Surgery	4,141	6	26.4	0.32 (0.13-0.80)	
		RAI	959	1[d]	17.7	1.00 (reference)	0.74
		Surgery	4,164	6	27.6	1.45 (0.17-12.42)	
	≥55	ATD	27,395	479	430.3	1.00 (reference)	0.60
		RAI	338	4	288.0	0.68 (0.16-2.89)	
		ATD	27,412	476	426.5	1.00 (reference)	0.003
		Surgery	1,957	13	144.3	0.34 (0.16-0.68)	
		RAI	333	2[d]	149.6	1.00 (reference)	0.72
		Surgery	1,986	10	119.7	0.77 (0.18-3.29)	
Cardiovascular mortality	20-54	ATD	87,617	132	34.3	1.00 (reference)	NA
		RAI	1,003	0	0.0	NA	
		ATD	87,595	131	34.0	1.00 (reference)	0.02
		Surgery	4,196	2[d]	8.4	0.25 (0.08-0.78)	
		RAI	991	0	0.0	1.00 (reference)	NA
		Surgery	4,220	4	17.2	NA	
	≥55	ATD	31,255	594	471.4	1.00 (reference)	0.56
		RAI	384	10	655.8	1.40 (0.45-4.34)	
		ATD	31,257	590	467.8	1.00 (reference)	0.09
		Surgery	2,161	26	276.9	0.59 (0.32-1.08)	
		RAI	376	11	718.8	1.00 (reference)	0.04
		Surgery	2,189	18	188.8	0.26 (0.07-0.95)	

MACE, major adverse cardiovascular events; ATD, anti-thyroid drug; RAI, radioactive iodine; IPTW, inverse probability of treatment weighting; NA, not applicable

[a] A pseudopopulation constructed by stabilized IPTW for analyses.

[b] In each outcome analysis, patients who had already experienced the corresponding outcome event before the index date were excluded.

© 2024 Peng CCH et al. *JAMA Network Open.*

[c] Per 100,000 person-years.

[d] In accordance with the data privacy protection regulation of the Ministry of Health and Welfare's Statistics Department, specific numbers cannot be disclosed when there are fewer than 3 events. However, the event number presented is calculated using inverse probability of treatment weighting, reflecting a weighted figure rather than the exact count of events.

© 2024 Peng CCH et al. *JAMA Network Open.*

eTable 10. Risks of MACE and all-cause mortality stratified by sex among patients with hyperthyroidism treated with ATD, RAI or surgery after IPTW[a]

Outcome[b]	Sex	Treatment	Patients, n	Event number	Incidence Rate[c]	HR (95% CI)	*p*-value
**MACE (composite outcome)**	Male	ATD	29,276	678	545.7	1.00 (reference)	0.08
		RAI	290	2[d]	149.2	0.27 (0.06-1.19)	
		ATD	29,270	689	554.5	1.00 (reference)	0.42
		Surgery	985	23	462.5	0.82 (0.51-1.32)	
		RAI	282	3[d]	199.9	1.00 (reference)	0.20
		Surgery	996	25	517.1	2.43 (0.63-9.39)	
	Female	ATD	77,769	1,050	310.7	1.00 (reference)	0.31
		RAI	941	9	202.2	0.65 (0.28-1.49)	
		ATD	77,779	1,059	313.6	1.00 (reference)	0.07
		Surgery	4,750	56	241.1	0.76 (0.57-1.02)	
		RAI	936	13	308.9	1.00 (reference)	0.78
		Surgery	4,783	61	265.4	0.84 (0.24-2.89)	
**All-cause mortality**	Male	ATD	33,119	937	670.4	1.00 (reference)	0.73
		RAI	323	8	578.4	0.86 (0.38-1.96)	
		ATD	33,112	948	678.6	1.00 (reference)	0.005
		Surgery	1,096	18	339.9	0.50 (0.31-0.81)	
		RAI	325	6	432.7	1.00 (reference)	0.51
		Surgery	1,105	31	592.4	1.36 (0.54-3.38)	
	Female	ATD	85,765	1,830	493.9	1.00 (reference)	0.06
		RAI	1,049	13	273.5	0.55 (0.30-1.04)	
		ATD	85,754	1,842	497.6	1.00 (reference)	<0.001
		Surgery	5,275	69	268.7	0.54 (0.40-0.72)	
		RAI	1,047	17	356.3	1.00 (reference)	0.80
		Surgery	5,304	80	318.1	0.89 (0.37-2.15)	

© 2024 Peng CCH et al. *JAMA Network Open.*

Outcome[b]	Sex	Treatment	Patients, n	Event number	Incidence Rate[c]	HR (95% CI)	*p*-value
**Acute myocardial infarction**	Male	ATD	32,710	156	113.1	1.00 (reference)	0.52
		RAI	324	1[d]	57.9	0.52 (0.07-3.75)	
		ATD	32,702	162	117.4	1.00 (reference)	0.26
		Surgery	1,086	3[d]	61.7	0.51 (0.16-1.64)	
		RAI	324	1[d]	68.7	1.00 (reference)	0.75
		Surgery	1,095	3[d]	53.0	0.70 (0.08-6.26)	
	Female	ATD	85,424	127	34.5	1.00 (reference)	0.82
		RAI	1,045	1[d]	27.4	0.80 (0.11-5.68)	
		ATD	85,417	128	34.8	1.00 (reference)	0.94
		Surgery	5,261	9	34.7	0.97 (0.43-2.19)	
		RAI	1,042	1[d]	15.9	1.00 (reference)	0.39
		Surgery	5,291	10	40.9	2.48 (0.31-19.63)	
**Stroke**	Male	ATD	30,872	361	275.8	1.00 (reference)	0.19
		RAI	304	1[d]	73.5	0.27 (0.04-1.9)	
		ATD	30,863	364	277.9	1.00 (reference)	0.73
		Surgery	1,022	16	312.4	1.10 (0.62-1.95)	
		RAI	304	1[d]	69.1	1.00 (reference)	0.14
		Surgery	1,034	18	358.3	4.72 (0.61-36.35)	
	Female	ATD	80,942	611	173.8	1.00 (reference)	1.00
		RAI	990	8	173.7	1.00 (0.44-2.28)	
		ATD	80,942	613	174.5	1.00 (reference)	0.34
		Surgery	4,937	50	207.8	1.17 (0.84-1.63)	
		RAI	987	13	302.5	1.00 (reference)	0.45
		Surgery	4,970	48	202.5	0.65 (0.21-2.01)	

© 2024 Peng CCH et al. *JAMA Network Open.*

Outcome[b]	Sex	Treatment	Patients, n	Event number	Incidence Rate[c]	HR (95% CI)	*p*-value
**Heart failure**	Male	ATD	31,326	285	214.6	1.00 (reference)	0.96
		RAI	307	3	224.6	1.06 (0.15-7.24)	
		ATD	31,322	286	215.8	1.00 (reference)	0.08
		Surgery	1,048	4	72.6	0.33 (0.10-1.12)	
		RAI	303	2[d]	150.6	1.00 (reference)	0.52
		Surgery	1,062	4	76.6	0.52 (0.07-3.82)	
	Female	ATD	81,806	495	139.6	1.00 (reference)	0.21
		RAI	991	3	57.3	0.41 (0.10-1.64)	
		ATD	81,808	500	141.1	1.00 (reference)	<0.001
		Surgery	5,052	12	46.9	0.33 (0.18-0.61)	
		RAI	987	2[d]	35.5	1.00 (reference)	0.73
		Surgery	5,088	12	50.4	1.30 (0.29-5.91)	
**Cardiovascular mortality**	Male	ATD	33,119	254	181.8	1.00 (reference)	0.46
		RAI	323	1[d]	86.7	0.48 (0.07-3.40)	
		ATD	33,112	261	186.5	1.00 (reference)	0.15
		Surgery	1,096	6	103.3	0.55 (0.25-1.23)	
		RAI	325	1[d]	72.6	1.00 (reference)	0.49
		Surgery	1,105	8	150.0	2.09 (0.25-17.31)	
	Female	ATD	85,765	472	127.3	1.00 (reference)	0.85
		RAI	1,049	5	114.4	0.90 (0.31-2.63)	
		ATD	85,754	475	128.3	1.00 (reference)	0.02
		Surgery	5,275	14	52.6	0.41 (0.19-0.86)	
		RAI	1,047	10	204.1	1.00 (reference)	0.05
		Surgery	5,304	12	46.5	0.22 (0.05-1.02)	

MACE, major adverse cardiovascular events; ATD, anti-thyroid drug; RAI, radioactive iodine; IPTW, inverse probability of treatment weighting

[a] A pseudopopulation constructed by stabilized IPTW for analyses

[b] In each outcome analysis, patients who had already experienced the corresponding outcome event before the index date were excluded.

© 2024 Peng CCH et al. *JAMA Network Open.*

[c] Per 100,000 person-years

[d] In accordance with the data privacy protection regulation of the Ministry of Health and Welfare's Statistics Department, specific numbers cannot be disclosed when there are fewer than 3 events. However, the event number presented is calculated using inverse probability of treatment weighting, reflecting a weighted figure rather than the exact count of events.

© 2024 Peng CCH et al. *JAMA Network Open.*

Table 11. Risks of MACE and all-cause mortality stratified by healthcare utilization among patients with hyperthyroidism treated with ATD, RAI or surgery after IPTW[a]

Outcome[b]	Healthcare utilization	Treatment	Patients, n	Event number	Incidence Rate[c]	HR (95% CI)	*p*-value
**MACE (composite outcome)**	Lower utilization[d]	ATD	56,187	729	299.7	1.00 (reference)	0.04
		RAI	608	1[e]	40.7	0.14 (0.02-0.93)	
		ATD	56,158	733	301.6	1.00 (reference)	0.006
		Surgery	2,937	23	154.6	0.51 (0.31-0.82)	
		RAI	607	1[e]	48.3	1.00 (reference)	0.06
		Surgery	2,961	24	163.7	3.22 (0.96-10.79)	
	Higher utilization[d]	ATD	50,845	999	456.3	1.00 (reference)	0.50
		RAI	623	10	343.5	0.75 (0.33-1.72)	
		ATD	50,872	1,006	459.5	1.00 (reference)	0.59
		Surgery	2,802	57	425.6	0.92 (0.68-1.24)	
		RAI	610	13	487.9	1.00 (reference)	0.92
		Surgery	2,822	62	470.8	0.94 (0.30-2.93)	
**All-cause mortality**	Lower utilization[d]	ATD	59,563	801	311.3	1.00 (reference)	0.26
		RAI	693	6	194.4	0.62(0.27-1.42)	
		ATD	59,510	814	316.6	1.00 (reference)	0.001
		Surgery	3,267	20	120.6	0.38 (0.21-0.66)	
		RAI	694	6	193.2	1.00 (reference)	0.75
		Surgery	3,293	26	159.2	0.83 (0.27-2.52)	
	Higher utilization[d]	ATD	59,302	1,966	777.4	1.00 (reference)	0.56
		RAI	685	19	626.4	0.81 (0.39-1.67)	
		ATD	59,314	1,974	781.2	1.00 (reference)	<0.001
		Surgery	3,093	68	463.7	0.59 (0.44-0.79)	
		RAI	679	22	739.7	1.00 (reference)	0.67
		Surgery	3,121	90	624.7	0.84 (0.38-1.87)	

© 2024 Peng CCH et al. *JAMA Network Open.*

Outcome[b]	Healthcare utilization	Treatment	Patients, n	Event number	Incidence Rate[c]	HR (95% CI)	*p*-value
**Acute myocardial infarction**	Lower utilization[d]	ATD	59,314	99	38.5	1.00 (reference)	NA
		RAI	688	0[e]	0.0	NA	
		ATD	59,261	99	38.5	1.00 (reference)	0.62
		Surgery	3,262	5	29.1	0.72 (0.20-2.59)	
		RAI	689	0[e]	0.0	1.00 (reference)	NA
		Surgery	3,289	4	22.5	NA	
	Higher utilization[d]	ATD	58,804	185	73.7	1.00 (reference)	0.99
		RAI	683	2[e]	74.2	1.01 (0.25-4.12)	
		ATD	58,815	188	75.0	1.00 (reference)	0.48
		Surgery	3,073	8	57.2	0.75 (0.33-1.68)	
		RAI	679	2[e]	56.9	1.00 (reference)	
		Surgery	3,100	9	64.7	1.12 (0.24-5.13)	0.88
**Stroke**	Lower utilization[d]	ATD	57,854	396	158.1	1.00 (reference)	0.13
		RAI	677	2[e]	59.6	0.38 (0.11-1.33)	
		ATD	57,826	396	158.4	1.00 (reference)	0.72
		Surgery	3,002	22	147.6	0.91 (0.55-1.52)	
		RAI	671	2[e]	73.8	1.00 (reference)	0.27
		Surgery	3,034	20	134.2	1.74 (0.65-4.67)	
	Higher utilization[d]	ATD	53,939	576	248.3	1.00 (reference)	0.85
		RAI	624	6	223.2	0.90 (0.29-2.77)	
		ATD	53,954	576	248.5	1.00 (reference)	0.22
		Surgery	2,956	45	317.6	1.26 (0.87-1.84)	
		RAI	615	11	383.7	1.00 (reference)	0.83
		Surgery	2,975	47	338.3	0.86 (0.21-3.54)	

© 2024 Peng CCH et al. *JAMA Network Open.*

Outcome[b]	Healthcare utilization	Treatment	Patients, n	Event number	Incidence Rate[c]	HR (95% CI)	*p*-value
**Heart failure**	Lower utilization[d]	ATD	57,780	305	122.1	1.00 (reference)	NA
		RAI	653	0[e]	0.0	NA	
		ATD	57,728	308	123.3	1.00 (reference)	<0.001
		Surgery	3,187	2[e]	11.9	0.09 (0.03-0.26)	
		RAI	655	0[e]	0.0	1.00 (reference)	NA
		Surgery	3,213	4	23.6	NA	
	Higher utilization[d]	ATD	55,341	475	200.1	1.00 (reference)	0.87
		RAI	645	5	181.3	0.91 (0.28-3.00)	
		ATD	55,367	477	201.0	1.00 (reference)	0.04
		Surgery	2,913	14	101.8	0.50 (0.26-0.96)	
		RAI	635	3	121.1	1.00 (reference)	0.64
		Surgery	2,940	13	93.2	0.75 (0.21-2.60)	
**Cardiovascular mortality**	Lower utilization[d]	ATD	59,563	222	86.2	1.00 (reference)	0.13
		RAI	693	1[e]	18.7	0.22(0.03-1.55)	
		ATD	59,510	226	87.8	1.00 (reference)	0.02
		Surgery	3,267	4	24.3	0.28 (0.10-0.79)	
		RAI	694	0[e]	15.2	1.00 (reference)	0.55
		Surgery	3,293	5	29.2	1.96 (0.22-17.45)	
	Higher utilization[d]	ATD	59,302	504	199.3	1.00 (reference)	0.93
		RAI	685	6	209.9	1.05 (0.35-3.22)	
		ATD	59,314	509	201.3	1.00 (reference)	0.08
		Surgery	3,093	16	110.3	0.55 (0.28-1.07)	
		RAI	679	12	395.0	1.00 (reference)	0.07
		Surgery	3,121	15	106.5	0.26 (0.06-1.09)	

MACE, major adverse cardiovascular events; ATD, anti-thyroid drug; RAI, radioactive iodine; IPTW, inverse probability of treatment weighting; NA, not applicable

[a] A pseudopopulation constructed by stabilized IPTW for analyses

[b] In each outcome analysis, patients who had already experienced the corresponding outcome event before the index date were excluded.

© 2024 Peng CCH et al. *JAMA Network Open.*

[c] Per 100,000 person-years

[d] Healthcare utilization was determined based on the number of clinic visits in the year preceding the index date. The lower group comprised patients with clinic visit numbers in the bottom 50%, while the higher group included those in the top 50%. Median [IQR] clinic visits for overall MACE, AMI, stroke, heart failure, cardiovascular mortality: ATD group 15 [15], RAI group 18 [16], surgery group 19 [17]

Median [IQR] clinic visits for all-cause mortality: ATD group 15 [16], RAI group 19 [17], surgery group 20 [19]

[e] In accordance with the data privacy protection regulation of the Ministry of Health and Welfare's Statistics Department, specific numbers cannot be disclosed when there are fewer than 3 events. However, the event number presented is calculated using inverse probability of treatment weighting, reflecting a weighted figure rather than the exact count of events

© 2024 Peng CCH et al. *JAMA Network Open.*

eTable 12. Risks of MACE and all-cause mortality stratified by index year among patients with hyperthyroidism treated with ATD, RAI or surgery after IPTW[a]

Outcome[b]	Index year	Treatment	Patients, n	Event number	Incidence Rate[c]	HR (95% CI)	*p*-value
**MACE (composite outcome)**	2011-2015	ATD	47,666	1,264	395.4	1.00 (reference)	0.06
		RAI	556	7	176.8	0.45 (0.20-1.02)	
		ATD	47,637	1,266	396.5	1.00 (reference)	0.34
		Surgery	3,029	71	345.0	0.87 (0.65-1.16)	
		RAI	552	8	216.8	1.00 (reference)	0.42
		Surgery	3,074	71	343.7	1.57 (0.52-4.70)	
	2016-2020	ATD	59,342	469	328.7	1.00 (reference)	0.14
		RAI	677	2[d]	115.1	0.35 (0.09-1.40)	
		ATD	59,371	473	331.0	1.00 (reference)	0.01
		Surgery	2,686	12	169.1	0.51 (0.30-0.86)	
		RAI	670	3	178.0	1.00 (reference)	0.64
		Surgery	2,707	17	238.7	1.34 (0.40-4.53)	
**All-cause mortality**	2011-2015	ATD	52,305	1,987	566.5	1.00 (reference)	0.17
		RAI	616	15	365.7	0.65 (0.35-1.21)	
		ATD	52,268	1,990	567.8	1.00 (reference)	<0.001
		Surgery	3,309	66	290.7	0.51 (0.38-0.68)	
		RAI	612	16	386.6	1.00 (reference)	0.97
		Surgery	3,356	86	381.2	0.99 (0.41-2.37)	
	2016-2020	ATD	66,542	786	492.0	1.00 (reference)	0.08
		RAI	766	4	218.8	0.44 (0.18-1.11)	
		ATD	66,556	792	496.1	1.00 (reference)	0.06
		Surgery	3,025	25	303.9	0.61 (0.36-1.03)	
		RAI	761	5	236.5	1.00 (reference)	0.29
		Surgery	3,059	30	379.0	1.62 (0.67-3.90)	

© 2024 Peng CCH et al. *JAMA Network Open.*

Outcome[b]	Index year	Treatment	Patients, n	Event number	Incidence Rate[c]	HR (95% CI)	*p*-value
**Acute myocardial infarction**	2011-2015	ATD	52,056	191	54.8	1.00 (reference)	0.55
		RAI	616	1[d]	29.6	0.55 (0.08-3.90)	
		ATD	52,020	190	54.6	1.00 (reference)	0.80
		Surgery	3,301	11	50.4	0.92 (0.48-1.77)	
		RAI	611	1[d]	17.8	1.00 (reference)	0.30
		Surgery	3,349	12	53.4	2.93 (0.38-22.64)	
	2016-2020	ATD	66,044	98	61.6	1.00 (reference)	0.92
		RAI	759	1[d]	55.5	0.90 (0.13-6.46)	
		ATD	66,055	98	61.7	1.00 (reference)	0.02
		Surgery	3,008	0[d]	6.0	0.09 (0.01-0.66)	
		RAI	755	1[d]	54.9	1.00 (reference)	0.25
		Surgery	3,042	1[d]	10.6	0.20 (0.01-3.09)	
**Stroke**	2011-2015	ATD	49,633	685	205.5	1.00 (reference)	0.73
		RAI	585	7	178.6	0.87 (0.40-1.91)	
		ATD	49,604	684	205.4	1.00 (reference)	0.10
		Surgery	3,135	58	271.2	1.32 (0.95-1.83)	
		RAI	580	10	261.6	1.00 (reference)	0.98
		Surgery	3,179	56	262.5	0.99 (0.32-3.07)	
	2016-2020	ATD	62,146	287	192.2	1.00 (reference)	NA
		RAI	717	0[d]	0.0	NA	
		ATD	62,170	289	193.4	1.00 (reference)	0.28
		Surgery	2,797	11	140.1	0.71 (0.38-1.33)	
		RAI	712	1[d]	28.8	1.00 (reference)	0.08
		Surgery	2,829	13	175.1	6.04 (0.78-46.51)	

© 2024 Peng CCH et al. *JAMA Network Open.*

Outcome[b]	Index year	Treatment	Patients, n	Event number	Incidence Rate[c]	HR (95% CI)	*p*-value
Heart failure	2011-2015	ATD	49,960	536	159.7	1.00 (reference)	0.13
		RAI	585	2[d]	54.3	0.34 (0.08-1.40)	
		ATD	49,924	538	160.2	1.00 (reference)	0.004
		Surgery	3,175	13	58.6	0.36 (0.18-0.73)	
		RAI	579	1[d]	34.4	1.00 (reference)	0.55
		Surgery	3,234	12	56.9	1.59 (0.35-7.17)	
	2016-2020	ATD	63,135	244	160.9	1.00 (reference)	0.99
		RAI	718	3	160.2	0.99 (0.15-6.58)	
		ATD	63,154	247	162.4	1.00 (reference)	0.004
		Surgery	2,897	3	35.5	0.21 (0.07-0.61)	
		RAI	715	2[d]	111.2	1.00 (reference)	0.42
		Surgery	2,918	4	47.4	0.43 (0.06-3.32)	
Cardiovascular mortality	2011-2015	ATD	52,305	525	149.8	1.00 (reference)	0.28
		RAI	616	3	76.3	0.51 (0.15-1.72)	
		ATD	52,268	526	150.2	1.00 (reference)	0.02
		Surgery	3,309	16	70.3	0.47 (0.25-0.89)	
		RAI	612	7	181.6	1.00 (reference)	0.29
		Surgery	3,356	16	70.4	0.39 (0.07-2.26)	
	2016-2020	ATD	66,542	205	128.6	1.00 (reference)	0.45
		RAI	766	1[d]	60.9	0.47 (0.07-3.37)	
		ATD	66,556	206	129.3	1.00 (reference)	0.09
		Surgery	3,025	4	52.2	0.40 (0.14-1.16)	
		RAI	761	1[d]	64.0	1.00 (reference)	0.93
		Surgery	3,059	5	57.7	0.91 (0.11-7.78)	

MACE, major adverse cardiovascular events; ATD, anti-thyroid drug; RAI, radioactive iodine; IPTW, inverse probability of treatment weighting; NA, not applicable

[a] A pseudopopulation constructed by stabilized IPTW for analyses

[b] In each outcome analysis, patients who had already experienced the corresponding outcome event before the index date were excluded.

© 2024 Peng CCH et al. *JAMA Network Open.*

[c] Per 100,000 person-years

[d] In accordance with the data privacy protection regulation of the Ministry of Health and Welfare's Statistics Department, specific numbers cannot be disclosed when there are fewer than 3 events. However, the event number presented is calculated using inverse probability of treatment weighting, reflecting a weighted figure rather than the exact count of events

© 2024 Peng CCH et al. *JAMA Network Open.*

eTable 13. Risks of hyperthyroidism relapse in patients treated with ATD, RAI, or surgery after IPTW[a]

Treatment	Patients, n	Event number	OR (95% CI)	*p*-value
ATD	119,182	75,607	1.00 (reference)	<0.001
RAI	1,410	601	0.43 (0.38-0.48)	
ATD	118,779	75,007	1.00 (reference)	<0.001
Surgery	6,871	848	0.08 (0.08-0.09)	
RAI	1,405	479	1.00 (reference)	<0.001
Surgery	6,938	1,317	0.45 (0.40-0.51)	

ATD, anti-thyroid drug; RAI, radioactive iodine; IPTW, inverse probability of treatment weighting; OR, odds ratio; CI, confidence interval

[a] A pseudopopulation constructed by stabilized IPTW for analyses

© 2024 Peng CCH et al. *JAMA Network Open.*

eTable 14. Risks of hyperthyroidism relapse stratified by age among patients with hyperthyroidism treated with ATD, RAI or surgery after IPTW[a]

Age group, y	Treatment	Patients, n	Event number	OR (95% CI)	*p*-value
20-54	ATD	87,858	57,123	1.00 (reference)	<0.001
	RAI	1,011	457	0.44 (0.39-0.50)	
	ATD	88,286	57,113	1.00 (reference)	<0.001
	Surgery	3,818	557	0.09 (0.09-0.10)	
	RAI	1,065	340	1.00 (reference)	<0.001
	Surgery	4,576	942	0.55 (0.48-0.64)	
≥ 55	ATD	31,314	18,467	1.00 (reference)	<0.001
	RAI	391	137	0.37 (0.30-0.46)	
	ATD	31,247	18,331	1.00 (reference)	<0.001
	Surgery	2,282	171	0.06 (0.05-0.07)	
	RAI	390	127	1.00 (reference)	<0.001
	Surgery	2,363	385	0.40 (0.32-0.51)	

ATD, anti-thyroid drug; RAI, radioactive iodine; IPTW, inverse probability of treatment weighting; OR, odds ratio; CI, confidence interval

[a] Patients who had both RAI and surgery were included in the analysis. A pseudopopulation constructed by stabilized IPTW for analyses

© 2024 Peng CCH et al. *JAMA Network Open.*

eTable 15.Risks of hyperthyroidism relapse stratified by sex among patients with hyperthyroidism treated with ATD, RAI or surgery after IPTW

Sex	Treatment	Patients, n	Event number	OR (95% CI)	*p*-value
Male	ATD	33,212	21,844	1.00 (reference)	<0.001
	RAI	324	158	0.49 (0.40-0.62)	
	ATD	33,190	21,763	1.00 (reference)	<0.001
	Surgery	1,066	130	0.07 (0.06-0.09)	
	RAI	357	132	1.00 (reference)	<0.001
	Surgery	1,181	221	0.39 (0.30-0.51)	
Female	ATD	85,980	53,755	1.00 (reference)	<0.001
	RAI	1,068	431	0.41 (0.36-0.46)	
	ATD	86,262	53,601	1.00 (reference)	<0.001
	Surgery	5,175	595	0.08 (0.07-0.09)	
	RAI	1,119	379	1.00 (reference)	<0.001
	Surgery	5,754	1,100	0.46 (0.40-0.53)	

ATD, anti-thyroid drug; RAI, radioactive iodine; IPTW, inverse probability of treatment weighting; OR, odds ratio; CI, confidence interval

[a] Patients who had both RAI and surgery were included in the analysis. A pseudopopulation constructed by stabilized IPTW for analyses

© 2024 Peng CCH et al. *JAMA Network Open.*

eTable 16. Sensitivity Analysis: Risks of MACE and all-cause mortality in hyperthyroid patients treated with ATD, RAI, or surgery, with landmark time (index date) at 24 months post-diagnosis after IPTW[a]

Outcome[b]	Treatment	Patients, n	Event number	Incidence rate[c]	HR (95% CI)	*p*-value
**MACE (composite outcome)**	ATD	100,389	1,539	373.4	1.00 (reference)	0.09
	RAI	1,192	10	204.1	0.55 (0.27-1.09)	
	ATD	100,391	1,552	376.6	1.00 (reference)	0.28
	Surgery	5,407	82	328.3	0.86 (0.66-1.12)	
	RAI	1,190	15	293.7	1.00 (reference)	0.96
	Surgery	5,536	77	310.3	1.02 (0.44-2.40)	
**All-cause mortality**	ATD	111,769	2,488	545.0	1.00 (reference)	0.31
	RAI	1,342	23	411.0	0.75 (0.44-1.31)	
	ATD	111,783	2,511	550.3	1.00 (reference)	0.003
	Surgery	6,025	98	355.2	0.64 (0.48-0.86)	
	RAI	1,336	24	439.5	1.00 (reference)	0.55
	Surgery	6,158	100	362.9	0.82 (0.42-1.60)	

MACE, major adverse cardiovascular events; ATD, anti-thyroid drug; RAI, radioactive iodine; IPTW, inverse probability of treatment weighting

[a] A pseudopopulation constructed by stabilized IPTW for analyses

[b] In each outcome analysis, patients who had already experienced the corresponding outcome event before the index date were excluded.

[c] Per 100,000 person-years

© 2024 Peng CCH et al. *JAMA Network Open.*

eTable 17. Sensitivity analysis after excluding incidental thyroid cancer[a]: Risks of MACE and all-cause mortality in patients with hyperthyroidism treated with ATD, RAI, or surgery after IPTW

Outcome[b]	Treatment	Patients, n	Event number	Incidence Rate[c]	HR (95% CI)	*p*-value
**MACE (composite outcome)**	ATD	106,236	1,701	371.0	1.00 (reference)	0.03
	RAI	830	6	162.8	0.44 (0.21-0.90)	
	ATD	106,239	1,712	373.5	1.00 (reference)	0.02
	Surgery	5,102	71	280.8	0.74 (0.58-0.96)	
	RAI	837	9	223.5	1.00 (reference)	0.57
	Surgery	5,138	79	314.4	1.36 (0.47-3.98)	
**All-cause mortality**	ATD	117,963	2,745	542.6	1.00 (reference)	0.08
	RAI	946	15	347.2	0.64 (0.39-1.05)	
	ATD	117,966	2,762	545.9	1.00 (reference)	<0.001
	Surgery	5,640	77	278.7	0.51 (0.39-0.66)	
	RAI	959	17	391.0	1.00 (reference)	0.50
	Surgery	5,676	84	304.5	0.76 (0.36-1.65)	
**Acute myocardial infarction**	ATD	117,218	277	55.1	1.00 (reference)	0.49
	RAI	941	1[d]	32.9	0.60 (0.15-2.51)	
	ATD	117,221	277	55.0	1.00 (reference)	0.33
	Surgery	5,619	11	40.9	0.72 (0.37-1.39)	
	RAI	954	1[d]	25.3	1.00 (reference)	0.48
	Surgery	5,657	12	45.2	1.70 (0.38-7.59)	
**Stroke**	ATD	110,961	963	201.4	1.00 (reference)	0.33
	RAI	890	5	136.0	0.68 (0.31-1.48)	
	ATD	110,964	967	202.1	1.00 (reference)	0.68
	Surgery	5,296	57	218.3	1.06 (0.79-1.43)	
	RAI	899	9	218.6	1.00 (reference)	0.95
	Surgery	5,336	61	234.0	1.04 (0.34-3.20)	
**Heart failure**	ATD	112,257	767	158.7	1.00 (reference)	0.19
	RAI	880	3	72.7	0.46 (0.15-1.46)	
	ATD	112,260	772	159.8	1.00 (reference)	<0.001
	Surgery	5,408	15	54.8	0.34 (0.19-0.61)	
	RAI	894	2[d]	53.0	1.00 (reference)	0.84
	Surgery	5,449	17	63.4	1.13 (0.33-3.81)	
**Cardiovascular mortality**	ATD	117,963	718	141.8	1.00 (reference)	0.44
	RAI	946	4	96.3	0.68 (0.26-1.80)	
	ATD	117,966	722	142.7	1.00 (reference)	0.007
	Surgery	5,640	18	66.6	0.46 (0.27-0.81)	
	RAI	959	8	185.1	1.00 (reference)	0.18

© 2024 Peng CCH et al. *JAMA Network Open.*

	Surgery	5,676	19	69.2	0.36 (0.08-1.62)

MACE, major adverse cardiovascular events; ATD, anti-thyroid drug; RAI, radioactive iodine; IPTW, inverse probability of treatment weighting; HR, hazard ratio; CI, confidence interval

[a] Incidental thyroid cancer was diagnosed during observation period.

[b] In each outcome analysis, patients who had already experienced the corresponding outcome event before the index date were excluded.

[c] Per 100,000 person-years

[d] In accordance with the data privacy protection regulation of the Ministry of Health and Welfare's Statistics Department, specific numbers cannot be disclosed when there are fewer than 3 events. However, the event number presented is calculated using inverse probability of treatment weighting, reflecting a weighted figure rather than the exact count of events

© 2024 Peng CCH et al. *JAMA Network Open.*

eTable 18. Sensitivity analysis using propensity score matching: Risks of MACE and all-cause mortality in patients with hyperthyroidism treated with ATD, RAI, or surgery

Outcome[a]	Treatment	Patients, n	Incidence rate[b]	HR (95% CI)	*p*-value
**MACE (composite outcome)**	ATD	911	314.2	1.00 (reference)	0.44
	RAI	911	216.7	0.67 (0.24-1.87)	
	ATD	5,311	418.2	1.00 (reference)	0.006
	Surgery	5,311	324.2	0.58 (0.39-0.86)	
	RAI	1,204	224.3	1.00 (reference)	0.15
	Surgery	1,204	508.8	1.88 (0.79-4.42)	
**All-cause mortality**	ATD	1,040	581.2	1.00 (reference)	0.29
	RAI	1,040	470.8	0.68 (0.34-1.39)	
	ATD	5,865	500.8	1.00 (reference)	0.004
	Surgery	5,865	316.4	0.60 (0.43-0.85)	
	RAI	1,356	435.0	1.00 (reference)	0.17
	Surgery	1,356	682.8	1.53 (0.83-2.82)	

MACE, major adverse cardiovascular events; ATD, anti-thyroid drug; RAI, radioactive iodine; IPTW, inverse probability of treatment weighting; HR, hazard ratio; CI, confidence interval

[a] In each outcome analysis, patients who had already experienced the corresponding outcome event before the index date were excluded.

[b] Per 100,000 person-years

© 2024 Peng CCH et al. *JAMA Network Open.*

eFigure 1. Illustrated study design

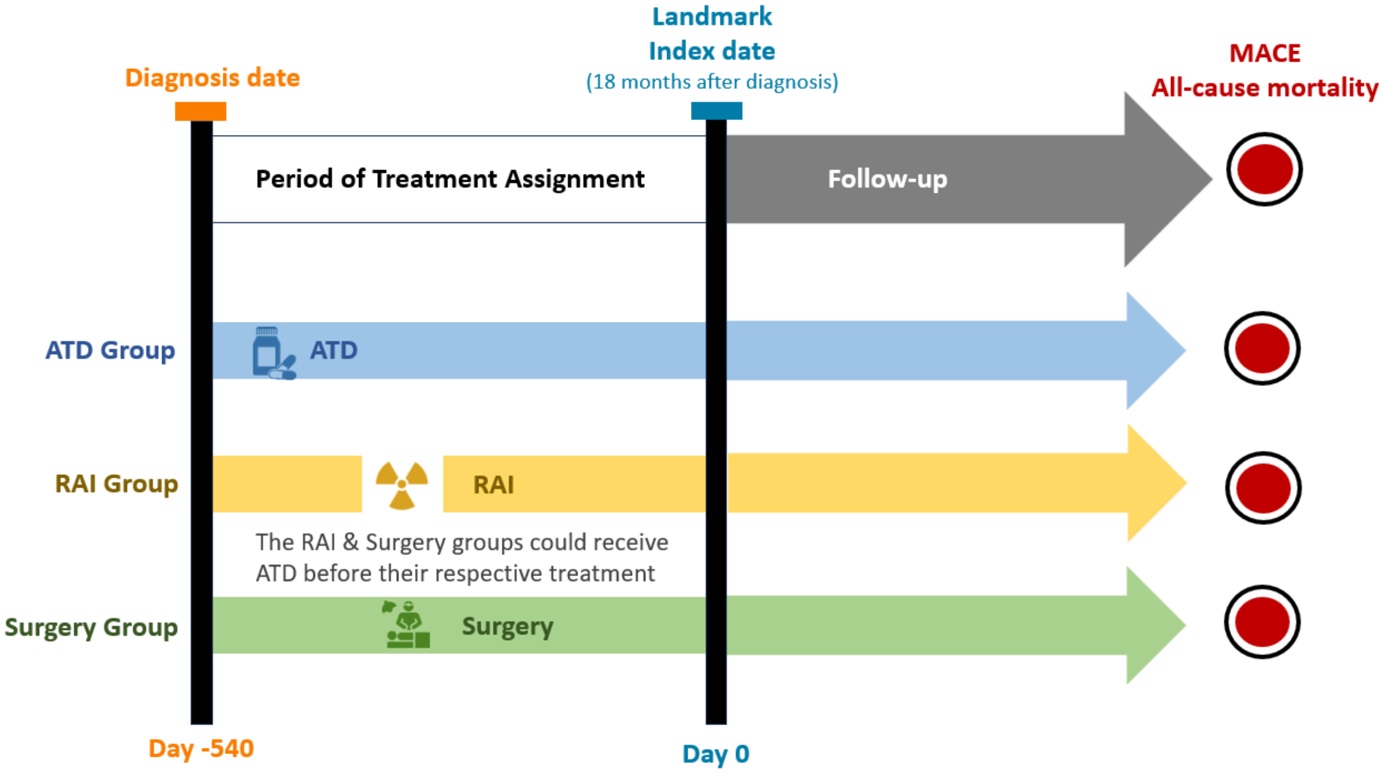

MACE, major adverse cardiovascular events; ATD, anti-thyroid drug; RAI, radioactive iodine

© 2024 Peng CCH et al. *JAMA Network Open.*

eFigure 2. The crude cumulative incidence curves of ATD, RAI, and surgery for (A) MACE and (B) all-cause mortality without IPTW

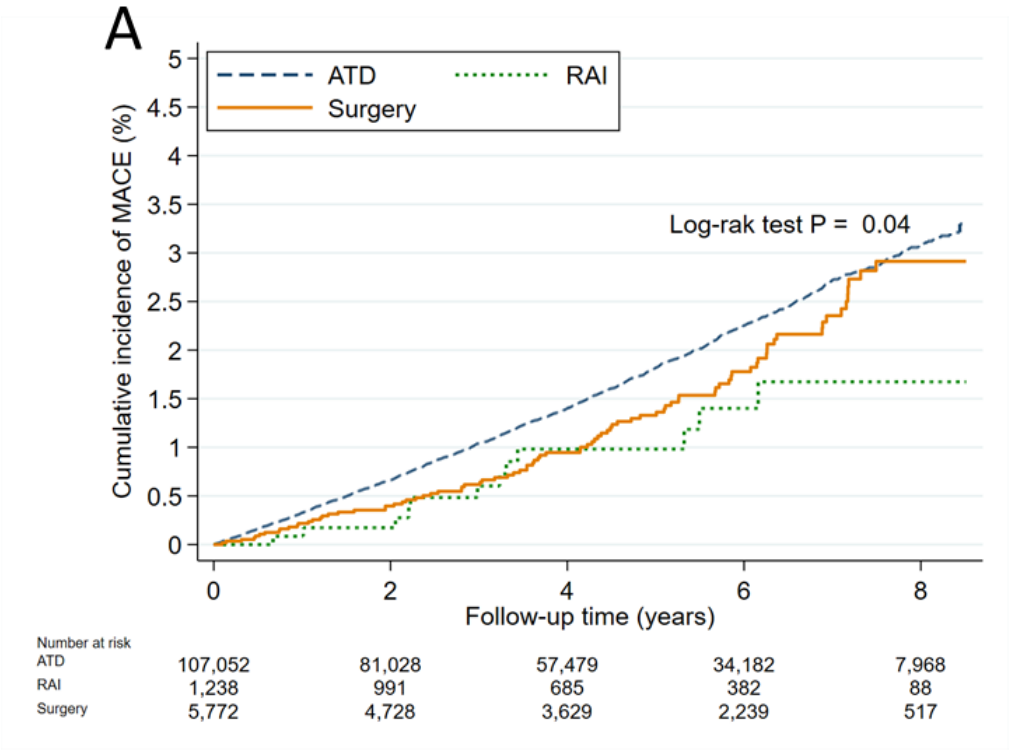

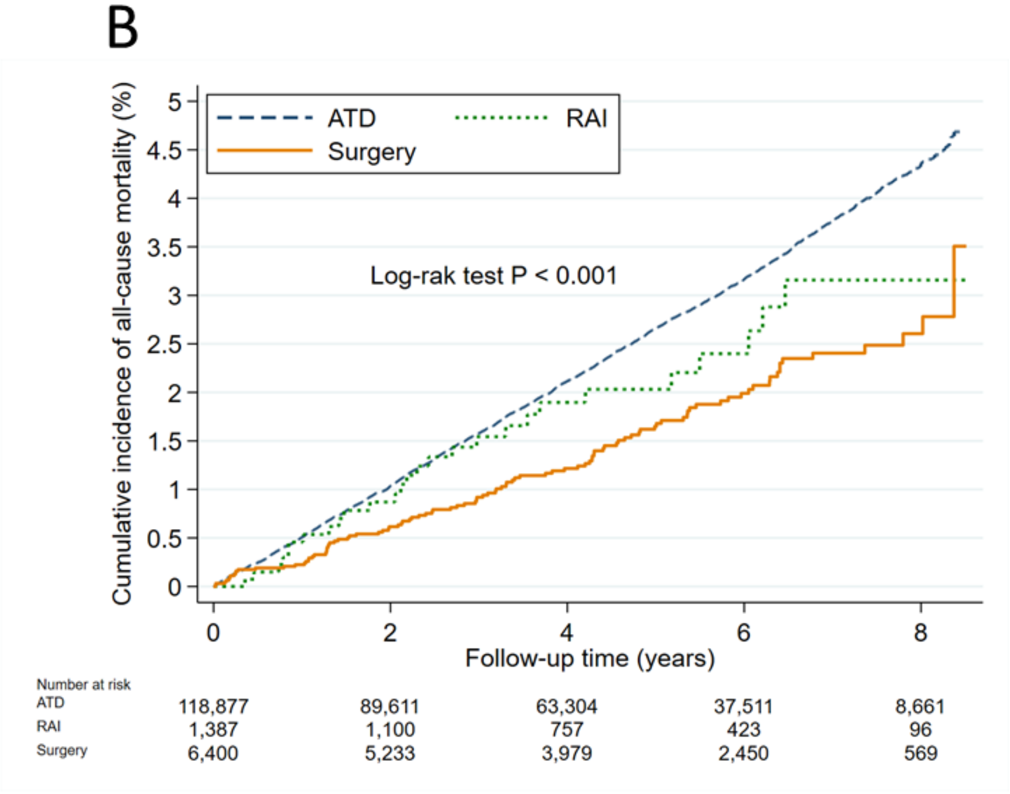

MACE, major adverse cardiovascular events; ATD, anti-thyroid drug; RAI, radioactive iodine

© 2024 Peng CCH et al. *JAMA Network Open.*

References

1. Morgan CJ. Landmark analysis: A primer. *J Nucl Cardiol*. Apr 2019;26(2):391-393. doi:10.1007/s12350-019-01624-z

2. Lévesque LE, Hanley JA, Kezouh A, Suissa S. Problem of immortal time bias in cohort studies: example using statins for preventing progression of diabetes. *Bmj*. Mar 12 2010;340:b5087. doi:10.1136/bmj.b5087

© 2024 Peng CCH et al. *JAMA Network Open.*
