# Supplementary material for: MACE and Hyperthyroidism Treated With Medication, Radioactive Iodine, or Thyroidectomy
Source: JAMA Netw Open. 2024 Mar 4;7(3):e240904. doi: 10.1001/jamanetworkopen.2024.0904 (PMC10912964; doi:10.1001/jamanetworkopen.2024.0904)
Supplement: Supplement 2. — Data Sharing Statement [file jamanetwopen-e240904-s002.pdf]

Data Sharing Statement

Peng. MACE and Hyperthyroidism Treated With Medication, Radioactive Iodine, or Thyroidectomy. *JAMA Netw Open*. Published online March 4, 2024. doi:10.1001/jamanetworkopen.2024.0904

Data
Data available: No

Additional Information
Explanation for why data not available: The datasets produced or analyzed in this study are not openly available because of the data protection regulations of the National Health Insurance Research Database (https://nhird.nhri.org.tw/en/Data_Protection.html). Researchers interested in examining these datasets must complete a formal application with the Taiwan Ministry of Health and Welfare to gain access. For additional details, please refer to their website at https://dep.mohw.gov.tw/DOS/cp-5119-59201-113.html .
